# A translational genomics approach identifies *IL10RB* as the top candidate gene target for COVID-19 susceptibility

**DOI:** 10.1038/s41525-022-00324-x

**Published:** 2022-09-05

**Authors:** Georgios Voloudakis, James M. Vicari, Sanan Venkatesh, Gabriel E. Hoffman, Kristina Dobrindt, Wen Zhang, Noam D. Beckmann, Christina A. Higgins, Stathis Argyriou, Shan Jiang, Daisy Hoagland, Lina Gao, André Corvelo, Kelly Cho, Kyung Min Lee, Jiantao Bian, Jennifer S. Lee, Sudha K. Iyengar, Shiuh-Wen Luoh, Schahram Akbarian, Robert Striker, Themistocles L. Assimes, Eric E. Schadt, Julie A. Lynch, Miriam Merad, Benjamin R. tenOever, Alexander W. Charney, Mount Sinai COVID-19 Biobank, VA Million Veteran Program COVID-19 Science Initiative, Kristen J. Brennand, John F. Fullard, Panos Roussos

**Affiliations:** 1 Department of Psychiatry, Icahn School of Medicine at Mount Sinai, New York, NY USA; 2 Center for Disease Neurogenomics, Icahn School of Medicine at Mount Sinai, New York, NY USA; 3 Pamela Sklar Division of Psychiatric Genomics, Icahn School of Medicine at Mount Sinai, New York, NY USA; 4 Friedman Brain Institute, Icahn School of Medicine at Mount Sinai, New York, NY USA; 5 Department of Genetics and Genomic Science, Icahn School of Medicine at Mount Sinai, New York, NY USA; 6 Icahn Institute for Data Science and Genomic Technology, Icahn School of Medicine at Mount Sinai, New York, NY USA; 7 Mental Illness Research, Education, and Clinical Center (VISN 2 South), James J. Peters VA Medical Center, Bronx, NY USA; 8 Nash Family Department of Neuroscience, Icahn School of Medicine at Mount Sinai, New York, NY USA; 9 Department of Psychiatry, Yale University, New Haven, CT USA; 10 Mount Sinai Clinical Intelligence Center, Icahn School of Medicine at Mount Sinai, New York, NY USA; 11 Department of Microbiology, Grossman School of Medicine, New York University, New York, NY USA; 12 Department of Medicine, Grossman School of Medicine, New York University, New York, NY USA; 13 Vilcek Institute of Graduate Biomedical Sciences, New York University, New York, NY USA; 14 Department of Microbiology, Icahn School of Medicine at Mount Sinai, New York, NY USA; 15 Virus Engineering Center for Therapeutics and Research, Icahn School of Medicine at Mount Sinai, New York, NY USA; 16 Global Health and Emerging Pathogens Institute, Icahn School of Medicine at Mount Sinai, New York, NY USA; 17 Biostatistics Shared Resources, Knight Cancer Institute, Oregon Health & Science University, Portland, OR USA; 18 VA Portland Health Care System, Portland, OR USA; 19 New York Genome Center, New York, NY USA; 20 VA Boston Healthcare System, Boston, MA USA; 21 Division of Aging, Brigham and Women’s Hospital, Harvard Medical School, Boston, MA USA; 22 VA Informatics and Computing Infrastructure, VA Salt Lake City Health Care System, Salt Lake City, UT USA; 23 Division of Epidemiology, University of Utah, Salt Lake City, UT USA; 24 Department of Medicine, Stanford University School of Medicine, Stanford, CA USA; 25 VA Palo Alto Health Care System, Palo Alto, CA USA; 26 Department of Population and Quantitative Health Sciences, School of Medicine, Case Western Reserve University, Cleveland, OH USA; 27 Department of Genetics and Genome Sciences, School of Medicine, Case Western Reserve University, Cleveland, OH USA; 28 VA Northeast Ohio Healthcare System, Cleveland VA Medical Center, Cleveland, OH USA; 29 Department of Medicine, Knight Cancer Institute, Oregon Health & Science University, Portland, OR USA; 30 Division of Infectious Diseases, Department of Medicine, University of Wisconsin, Madison, WI USA; 31 William S. Middleton Memorial Veterans Hospital, Madison, WI USA; 32 Sema4, Stamford, CT USA; 33 Department of Oncological Sciences, Icahn School of Medicine at Mount Sinai, New York, NY USA; 34 Precision Immunology Institute, Icahn School of Medicine at Mount Sinai, New York, NY USA; 35 Tisch Cancer Institute, Icahn School of Medicine at Mount Sinai, New York, NY USA

**Keywords:** Gene expression, Infectious diseases

## Abstract

Recent efforts have identified genetic loci that are associated with coronavirus disease 2019 (COVID-19) infection rates and disease outcome severity. Translating these genetic findings into druggable genes that reduce COVID-19 host susceptibility is a critical next step. Using a translational genomics approach that integrates COVID-19 genetic susceptibility variants, multi-tissue genetically regulated gene expression (GReX), and perturbagen signatures, we identified *IL10RB* as the top candidate gene target for COVID-19 host susceptibility. In a series of validation steps, we show that predicted GReX upregulation of *IL10RB* and higher *IL10RB* expression in COVID-19 patient blood is associated with worse COVID-19 outcomes and that in vitro *IL10RB* overexpression is associated with increased viral load and activation of disease-relevant molecular pathways.

## Introduction

Severe acute respiratory syndrome coronavirus 2 (SARS-CoV-2), which causes coronavirus disease 2019 (COVID-19), is the latest of the betacoronaviruses to pose a global health threat. Of the recent respiratory virus pandemics, SARS-CoV-2 demonstrates the highest transmissibility^1^. Despite the fact that the overwhelming majority of affected individuals have mild symptoms, infection-fatality risk in an urban area of a developed country (e.g., New York City) is still high, ranging from 1.4% for young adults (25–44 years old) to 19.1% for more susceptible older individuals (aged 75 years and older)^2^. Unexplained heterogeneity in susceptibility to the disease and severity of illness exists, even when accounting for known risk factors such as age^3^.

The COVID-19 Host Genetics Initiative (HGI)^4,5^ coordinates a global effort to elucidate the genetic basis of COVID-19 susceptibility. Ongoing efforts have uncovered multiple risk loci for COVID-19 susceptibility; however, these risk variants only partly explain interindividual variability and, as many of the variants reside within noncoding regions of the genome, the formulation of testable hypotheses to elucidate their potential effects is challenging. To translate these genetic findings into novel therapeutics for COVID-19, we sought to prioritize druggable gene targets by developing a multidisciplinary translational genomics framework that integrates genetic studies of COVID-19 susceptibility, genotype-tissue expression datasets, and perturbagen signature libraries. We provide evidence from in vitro, in vivo, and retrospective epidemiological studies that validate the association of the top candidate gene, interleukin 10 receptor subunit beta (*IL10RB*), with COVID-19 outcome severity. Overall, our study identified gene targets with direct translational value to modulate host physiology and immune response, and increase resilience to SARS-CoV-2 infection.

## Results

### Overview of the multidisciplinary translational genomics framework

**Fig. 1:**
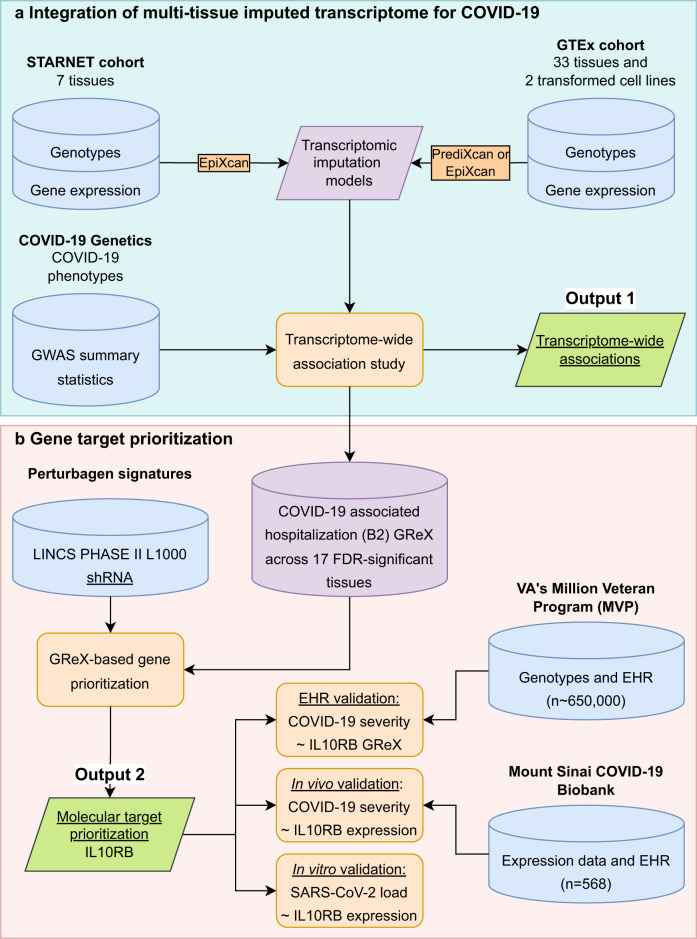
Data-driven GReX (genetically regulated gene expression)-based approach for molecular target prioritization for COVID-19. **a** Multi-tissue (*n* = 42) transcriptome-wide association study using: (1) GWAS summary statistics from the COVID-19 Host Genetics Initiative for COVID-19 phenotypes, and (2) transcriptomic imputation models trained in the STARNET (Stockholm-Tartu Atherosclerosis Reverse Network Engineering Task) and GTEx (Genotype-Tissue Expression) cohorts. **b** Gene target prioritization via integration of multi-tissue transcriptomes (17 FDR-significant tissues for COVID-19 associated hospitalization) with perturbational transcriptomic profiles from LINCS (library of integrated network-based cellular signatures) identified *IL10RB* as the top candidate. In a series of validation experiments we found that: (1) blood *IL10RB* genetically regulated gene expression (GReX) is associated with COVID-19 severity in the VA’s Million Veteran Program—“EHR validation”, (2) COVID-19 severity was associated with increased assayed *IL10RB* expression in patients’ blood—“in vivo validation”, and (3) increasing *IL10RB* expression resulted in higher SARS-CoV-2 viral load in two different model cell systems for SARS-CoV-2 infection and replication—“in vitro validation”.

We developed a translational genomics framework that integrates three major sources of data (GWAS, genotype-tissue expression datasets, and perturbagen signature libraries) to identify and validate susceptibility genes for targeted therapeutics (Fig. 1). We first integrated GWASs for COVID-19 phenotypes with multi-tissue transcriptomic imputation models to predict genetically regulated gene expression (GReX) changes associated with COVID-19 susceptibility (Fig. 1a; Output 1).

We developed and applied a gene prioritization approach (Fig. 1b; Output 2) that integrates GReX with shRNA signature libraries^6^ to identify key genes whose expression: (a) is predicted to be dysregulated in COVID-19 susceptible individuals, and (b) can be targeted to reverse the transcriptome-wide gene expression dysregulation that is associated with COVID-19 susceptibility. From this prioritization step, we identified *IL10RB* as the top gene target candidate, which we then subjected to three validation steps. The first validation step was to increase phenotypic specificity by examining whether *IL10RB* GReX is associated with COVID-19 severity in patients that tested positive for SARS-CoV-2 (Fig. 1b: EHR validation). In addition, we performed a GReX-based phenome-wide association study (PheWAS) to further understand the effect of *IL10RB* on pre-existing relevant phenotypes (before the emergence of COVID-19). The second validation step sought to associate *IL10RB* gene expression in peripheral blood with COVID-19 severity in a patient cohort (Fig. 1b: in vivo validation). The third, and final, validation step involved isogenic manipulation of *IL10RB* gene expression in vitro to study its effect on SARS-CoV-2 viral load and transcriptional dysregulation (Fig. 1b: in vitro validation).

### COVID-19 transcriptome-wide association study

We performed a transcriptome-wide association study (TWAS) leveraging 2 transformed cell lines and 40 peripheral tissue models from two cohorts (GTEx: Genotype-Tissue Expression v8^7^ and STARNET: Stockholm-Tartu Atherosclerosis Reverse Network Engineering Task^8^; *n* = 16,738 reliably imputed genes; Supplementary Table 1) by using GWAS summary statistics for COVID-19 phenotypes^4^ (Supplementary Table 2). Overall, for COVID-19 phenotypes, we observed a high correlation among the imputed transcriptomes of different tissues (Supplementary Fig. 1), even though the imputed transcriptomes of the COVID-19 phenotypes are quite diverse (Supplementary Fig. 2; a more detailed description of the different phenotypes can be found in the Supplementary Information). 17 genes were significantly associated with COVID-19 infection and outcomes (FDR-adjusted p ≤ 0.05) when considering all COVID-19 phenotypes and 42 tissues: *CCR1, CCR2, CCR3, CCR5, CXCR6, DNPH1, DPP9, IFNAR2, IL10RB, IL10RB-AS1, KCNN3, KIF15, OAS1, OAS2, OAS3, PDE4A,* and *TMEM241*. Some of these genes were identified in more than one COVID-19 phenotype (e.g., *IL10RB* and *IFNAR2*), whereas others (e.g., *OAS2*) were only associated with one (Supplementary Fig. 3); the GWAS contributing the highest number of gene-trait associations is “hospitalized COVID vs. population” (B2 phenotype as per COVID-19 HGI; 13 out of the 17 genes are captured; Supplementary Fig. 3). We also observed that most gene-trait associations are detected in Blood (STARNET), and Mammary artery (STARNET), identifying 7 and 6 gene-trait associations, respectively (Supplementary Fig. 4). For the remainder of our analysis we thus focused on the B2 phenotype which we will refer to as “COVID-19 associated hospitalization” as: (1) it is the highest powered GWAS, and (2) conceptually, it captures genetic determinants protecting individuals both from infection (since the control group could have been SARS-CoV-2 negative or nonhospitalized positive) and from severe outcomes of COVID-19. Unsurprisingly, B2 exhibits moderate GReX correlation with both these phenotypes (Supplementary Fig. 2).

**Fig. 2:**
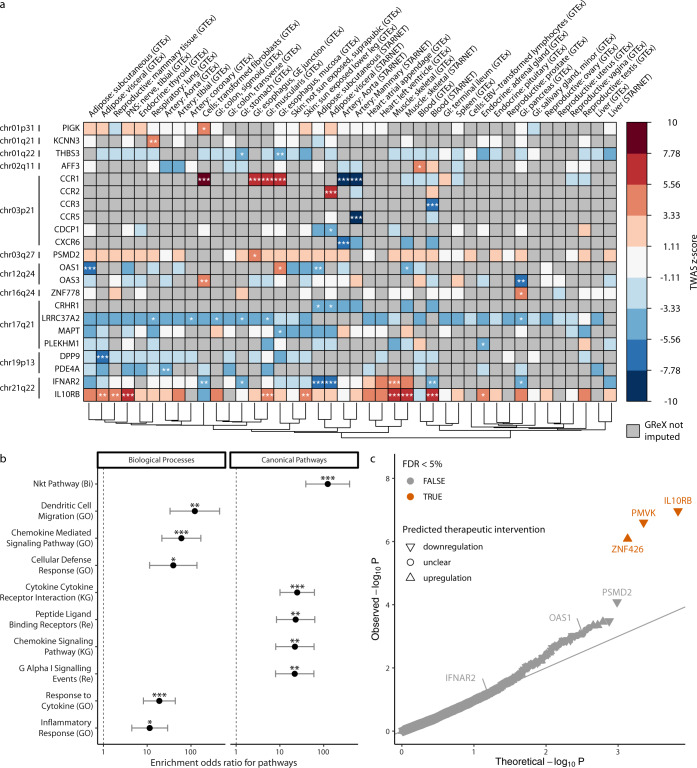
Transcriptome-Wide Association Study (TWAS) for COVID-19-associated hospitalization (hospitalized COVID vs. the general population) identifies associated genes, pathways, and aids in identification of druggable gene targets. **a** FDR-significant TWAS results for COVID-19 susceptibility across all tissues. Box color indicates gene-trait-tissue association z-scores. Gray squares represent genes whose genetically regulated gene expression (GReX) could not be imputed. ***, **, and * correspond to FDR-adjusted *p*-values of association equal or less than 0.001, 0.01, and 0.05 respectively. Dendrogram on the bottom edge is shown from Ward hierarchical clustering for tissues based on all GReX (not just FDR-significant results). Displayed results are limited to protein-coding genes; cytogenetic location (at band level resolution) is also provided on the left of each gene. **b** Enrichment of COVID-19 TWAS associated genes for biological processes and canonical pathways. Odds ratio with 95% confidence interval (CI) is plotted for the significant enrichments of TWAS gene-trait associations from all tissues. Pathways are ranked based on estimated enrichment odds ratio. Analysis is limited to protein-coding genes and excludes genes residing in the major histocompatibility complex (MHC) on chromosome 6. Enrichments that are FDR significant are annotated as follows: *, **, and *** for FDR-adjusted *p* ≤ 0.05, 0.01, and 0.001 respectively; Fisher’s exact test. **c** Prioritization of candidate gene targets to reverse TWAS gene-trait associations. *p*-value is estimated based on the joint statistic of two approaches ($z_{{\mathrm{combined}}} = \underline {z_{TWAS}} + {\mathrm{pseudo}}\;z_{GReX\;{\mathrm{antagonism}}}$) against the null. FDR-significant candidate genes are labeled orange. The direction of the predicted therapeutic intervention (upregulation or downregulation) is illustrated. IL10RB, PMVK, and ZNF426 are FDR-significant target genes (*n* = 4302 imputed genes with reliable shRNA signatures).

When considering only the COVID-19-associated hospitalization (B2) phenotype, we identified 88 gene-trait-tissue associations corresponding to 26 unique gene-trait associations (*AFF3, CCR1, CCR2, CCR3, CCR5, CDCP1, CRHR1, CXCR6, DPP9, IFNAR2, IL10RB, IL10RB-AS1, KANSL1-AS1, KCNN3, LINC02210, LRRC37A2, LRRC37A4P, MAPT, OAS1, OAS3, PDE4A, PIGK, PLEKHM1, PSMD2, THBS3, ZNF778*; FDR-adjusted *p* ≤ 0.05 while only considering B2; Supplementary Data 1–7 for all results split by COVID-19 phenotype) across 11 genomic regions (Fig. 2a for protein-coding genes). Testing a multitude of tissues across different cohorts allowed us to detect the consistent patterns of GReX dysregulation; for example, *IL10RB* is FDR significant in nine different tissue models (Fig. 2a). Significant genes (from at least one tissue) are enriched for pathways mainly involved in immune host response (Fig. 2b); a finding which is replicated when employing an LD-aware, competitive pathway TWAS method (JEPEGMIX2-P^9^; Supplementary Data 8). Overall, these results indicate that genetically-associated changes in genes involved in immune-related pathways predispose individuals to more severe COVID-19 outcomes.

### Gene target prioritization identifies *IL10RB* as a key regulator

Towards prioritizing genes as putative molecular targets for intervention, we next aimed to identify genes whose perturbation is predicted to be therapeutic by antagonizing the GReX associated with COVID-19 susceptibility. Using a computational shRNA antagonism approach^10,11^ (Supplementary Fig. 5), whose output we integrated with the TWAS findings, we identified *IL10RB* as the top most significant candidate for gene targeting (Fig. 2c). *IL10RB* was predicted to be significantly upregulated in individuals susceptible to COVID-19 hospitalization, with downregulation predicted to significantly antagonize the polygenetically driven gene expression differences associated with COVID-19 hospitalization (Supplementary Fig. 5). In mouse models, IL10RB overexpression was shown to increase susceptibility to lethal bacterial superinfections in the lungs via postviral increased IL-10 signaling, which dampens the immune response^12^, and by direct disruption of the lung epithelial barrier through increased expression of type III interferons (IFNλ)^13^. *IFNAR2*, which is FDR significant in the TWAS and in the same locus as *IL10RB* (less than 2kbp from *IL10RB*), was not significant in this analysis, thereby nominating *IL10RB* as the most promising candidate in the locus while deprioritizing *IFNAR2*. Since this was a new approach, we tested both *IL10RB* and *IFNAR2* in our downstream analyses to determine how well our prioritization strategy performed in differentiating candidate genes in such close proximity to one another.

### Predicted upregulation of blood *IL10RB* is associated with COVID-19 severity and increased incidence of respiratory failure

**Fig. 3:**
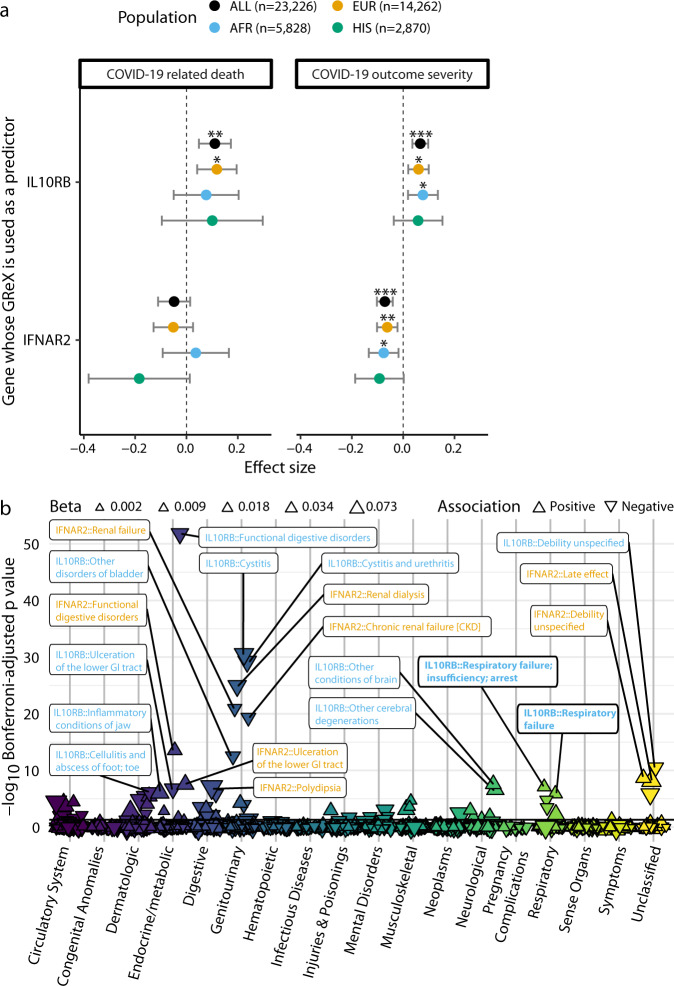
Association of blood *IL10RB* and *IFNAR2* genetically regulated gene expression (GReX) with COVID-19-related outcomes and non-COVID-19 phenotypes. **a** GReX of *IL10RB* and *IFNAR2* was imputed in 23,216 individuals in the Million Veteran Program (MVP) cohort for whom COVID-19 outcome severity information was available. For COVID-19-related death (left panel) we checked the association of GReX with the outcome of COVID-19 related death (4.8% of this cohort) under logistic regression models for *IL10RB* and *IFNAR2* GReX, while adjusting for age, sex, Elixhauser’s comorbidity score, and ancestry-specific population structure. For COVID-19 outcome severity, we applied an ordinal regression model (same predictors and covariates as above) using an outcome scale corresponding to mild (74.9% of the cohort), moderate (17%), severe (3.2%) COVID-19 related outcomes, and death (4.8%). EUR, AFR, and HIS refer to harmonized European, African and Hispanic ancestry respectively and the sample sizes are provided in the legend at the top. For both panels, a population of Asian ancestry (*n* = 266) was included in the fixed effects meta-analysis (Population: “ALL” in the graph) but not plotted. ***, **, and * correspond to Bonferroni-adjusted association *p*-values (for n_genes_ × n_outcomes_ for each population cohort) of equal or less than 0.001, 0.01, and 0.05 respectively. Error bars show 95% CI. **b** Phenome-wide association study (PheWAS) of *IL10RB* and *IFNAR2* blood GReX for individuals of European descent in the MVP cohort (*n* = 296,407). Phenotypes are grouped in categories shown in the *x*-axis, while the *y*-axis represents −log_10_(Bonferroni-adjusted *p*-values). Triangles represent data points for positive (pointing up) and negative (pointing down) association with GReX; triangle size indicates the magnitude of the effect size and the color corresponds to the phenotype category. Only the top 20 associations are labeled (orange for *IFNAR2* and blue for *IL10RB*); full results are provided in Supplementary Data 9. The horizontal black line corresponds to Bonferroni-adjusted *p* = 0.05.

The COVID-19-related hospitalization GWAS utilized a broadly-defined phenotype to increase cohort inclusion and sample size. To enhance granularity and phenotyping depth of *IL10RB* GReX association with COVID-19 associated hospitalization, we determined whether predicted upregulation of blood *IL10RB* was a good predictor of COVID-19 outcome severity and death in individuals who tested positive for SARS-CoV-2. We performed individual GReX imputation and association analysis in the VA’s Million Veteran Program (MVP)^14^, where the severity of COVID-19-related outcomes was deduced from EHR of COVID-19 positive cases (*n* = 23,226; cohort characteristics in Supplementary Table 3). *IL10RB* GReX was associated with increased incidence of COVID-19 related death in individuals of European descent (EUR; logistic regression; OR = 1.13; Bonferroni-adjusted *p* = 0.01; *n* = 14,262) and in the trans-ethnic meta-analysis (logistic regression; OR = 1.12; Bonferroni-adjusted *p* = 0.002; *n* = 23,226) (Fig. 3a and Supplementary Fig. 6); *IFNAR2* GReX was not associated with COVID-19 death. However, both *IL10RB* and *IFNAR2* GReX are associated with more severe COVID-19 clinical outcomes in EUR, participants of African descent (AFR), and in the trans-ethnic meta-analyses (ordinal logistic regression; Fig. 3a and Supplementary Fig. 6; Supplementary Table 4). It is important to note the association with severity or death controls for age, sex, Elixhauser’s comorbidity score^15^, and ancestry-specific population structure (Fig. 3a).

To better understand the phenotypic variation linked with *IL10RB* and *IFNAR2* imputed expression, we next performed a phenome-wide association study (PheWAS) utilizing the *IL10RB* and *IFNAR2* GReX models in MVP (Fig. 3b; cohort characteristics in Supplementary Table 3; Supplementary Data 9 for a complete set of results). For *IL10RB*, among significant results, we found that COVID-19-related GReX dysregulation (higher *IL10RB* GReX in the blood) was positively associated with respiratory failure and tracheostomy complications and disorders of the circulatory system such as heart aneurysms and non-rheumatic mitral valve disorders, as well as cholecystitis without cholelithiasis and inflammatory conditions of the jaw. On the other hand, *IL10RB* was negatively associated with intracerebral hemorrhage, infections of the skin (e.g., lower limb cellulitis) and genitourinary system (e.g., cystitis and urethritis), type 1 diabetes, kidney disease (e.g., renal osteodystrophy), schizophrenia, functional disorders of the digestive system and bladder, and overall unspecified debility and sequela. COVID-19-related *IFNAR2* GReX dysregulation (lower *IFNAR2* GReX in the blood) shares some positive associations with *IL10RB, including* respiratory failure and heart aneurysms, but is independently associated with congestive heart failure, (chronic) renal failure and dialysis, delirium dementia, stomach cancer, and antisocial/borderline personality disorder. Furthermore, we also identified negative associations with cerebral ischemia, specific infections (cellulitis of foot, toe, and pyoderma, acute osteomyelitis), arthropathies, functional disorders of the digestive system and bladder, and overall unspecified debility and sequela. Overall, in addition to predisposing individuals to COVID-19-related hospitalization and outcome severity, increased *IL10RB* and decreased *IFNAR2* GReX are associated with respiratory failure independent of COVID-19 exposure.

### Increased *IL10RB* blood expression predicts worse COVID-19 outcome

**Fig. 4:**
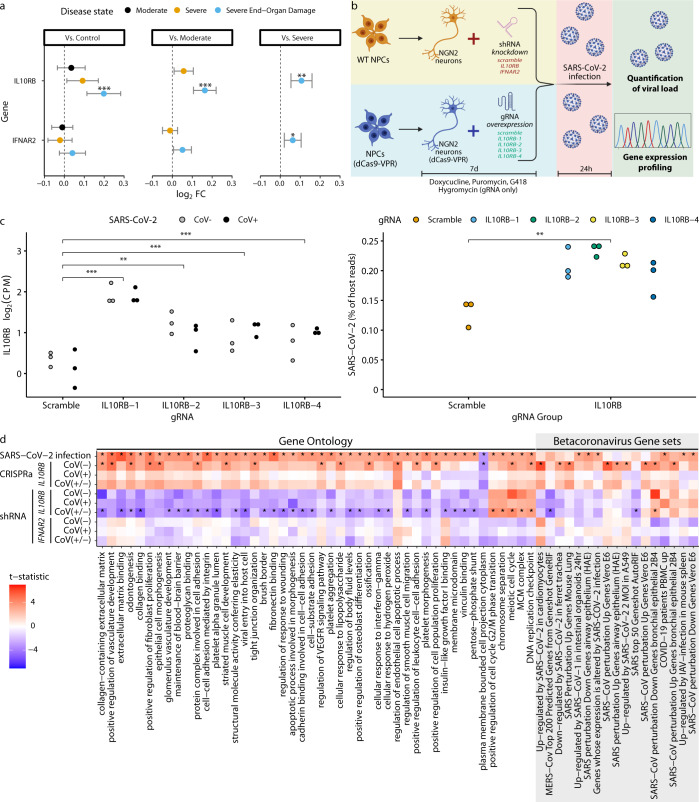
Increased *IL10RB* expression is associated with worse COVID-19 outcomes in vivo and increased SARS-CoV-2 viral load in vitro. **a** Increased *IL10RB* expression is associated with worse COVID-19 outcomes in vivo. *, **, and *** for FDR-adjusted *p* (FDR) ≤ 0.05, 0.01, and 0.001, respectively. Error bars show 95% CI. **b** In vitro experimental overview. **c** CRISPRa gRNAs (IL10RB-1, IL10RB-2, IL10RB-3, and IL10RB-4) were used to overexpress *IL10RB* in hiPSC-derived NGN2-glutamatergic neurons. ***, **, and * correspond to *p-*values from the linear model of equal or less than 0.001, 0.01, and 0.05, respectively. For the SARS-CoV-2 viral load (right panel) we performed pairwise comparison with unpaired *t*-test; ***, **, and * correspond to *p* values equal to, or less than, 0.001, 0.01, and 0.05, respectively. **d** Competitive gene set enrichment analysis in hiPSC-derived NGN2 glutamatergic neurons. Each row represents a different experimental condition and each column a different gene set; the top row shows the effect of SARS-CoV-2 infection, while the remaining rows show the effect of gene manipulation (e.g., IL10RB vs. nontargeting siRNA) within a specific group (e.g., CoV(−): uninfected cells). The left side of the plot (Gene ontology gene sets; white background) indicates enrichment for canonical pathways and biological processes that are significantly enriched (FDR < 0.05) in SARS-CoV-2 infection (top row), while the right side (Betacoronavirus Gene sets; gray background) illustrates enrichment for gene sets that correspond to betacoronavirus infections across different cell systems^21^ (only significant results are shown; FDR < 0.05).

Transcriptome imputation models can only partially explain the variance in observed *IL10RB* and *IFNAR2* gene expression (R^2^_CV_ is 0.099 and 0.278, respectively). To further confirm the association of IL10RB with COVID-19 severity, we utilized blood gene expression profiling data from COVID-19 patients and controls at the Mount Sinai COVID-19 Biobank^16^ (cohort characteristics in Supplementary Table 5 and Supplementary Table 6). We established a direct significant association between observed blood *IL10RB* expression and severe COVID-19 outcomes, including end-organ damage. The levels of *IL10RB* expression gradually increased with disease severity with a higher effect size in the most severe COVID-19 patient group (end-organ damage) against all other groups (Fig. 4a). Similar analysis for blood *IFNAR2* gene expression failed to demonstrate a robust association.

### *IL10RB* overexpression increases in vitro SARS-CoV-2 viral load

It has recently been shown that SARS-CoV-2 viral load in patients is associated with increased disease severity and mortality^17^. To explore the effect of *IL10RB* expression on SARS-CoV-2 viral load, we performed a series of in vitro experiments where we manipulated gene expression levels with a short hairpin (shRNA; for downregulation) and clustered regularly interspaced short palindromic repeats activation (CRISPRa; for upregulation) and quantified SARS-CoV-2 viral load (Fig. 4b). Overall, we tested (in technical triplicates) downregulation of *IL10RB* (and *IFNAR2*) by shRNA (Supplementary Fig. 7; Supplementary Fig. 8) and upregulation of *IL10RB* by CRISPRa (by using four different guide RNAs; Fig. 4c). We performed these experiments in NGN2-glutamatergic postmitotic neurons^18^ derived from human induced pluripotent stem cells (hiPSCs). These cells are permissive to SARS-CoV-2 and, therefore, can serve as a model cell system for SARS-CoV-2 infection^19^. Importantly, for the same COVID-19 phenotype (B2), *IL10RB* brain GReX has the same direction of predicted dysregulation (z-score = 5.53; dorso-lateral prefrontal cortex)^20^ as the STARNET blood (z-score = 6.06) and GTEx lung (z-score = 3.02) models in this study. Indeed, we found that the gene expression dysregulation caused by SARS-CoV-2 infection in our model cell system mimics the transcriptional signatures corresponding to SARS-CoV-2 (and other betacoronaviruses) infection of a diverse range of cell types^21^ (Fig. 4d; Supplementary Fig. 9).

We observed a significant increase in SARS-CoV-2 viral load (Fig. 4c; *p* = 0.0087; unpaired *t*-test) after *IL10RB* overexpression using four different guide RNAs (gRNAs) (Fig. 4c and Supplementary Table 7). Competitive pathway enrichment analysis demonstrated that overexpressing *IL10RB* in uninfected cells leads to the induction of COVID-19 relevant pathways implicated in vascular, immune system, and extracellular matrix processes (Fig. 4d), which were also activated by SARS-CoV-2 infection (Fig. 4d). Surprisingly, even in the absence of SARS-CoV-2, *IL10RB* overexpression leads to transcriptional changes reminiscent of betacoronavirus infection (Supplementary Fig. 9). In the rescue experiment, shRNA knockdown of *IL10RB* did not reduce *IL10RB* levels robustly, most likely due to low basal expression (Supplementary Fig. 7; Supplementary Table 8); it is worth noting that basal *IL10RB* expression has been shown to be low in all cell types from lung, heart, liver, and kidney^22^. We were also able to successfully knock down *IFNAR2* (higher basal expression; Supplementary Fig. 8; Supplementary Table 8), leading to a decrease in SARS-CoV-2 load. Interestingly, SARS-CoV-2 infection-induced expression of *IFNAR2* (Supplementary Fig. 8; Supplementary Table 9) but not *IL10RB* (Supplementary Fig. 7; Supplementary Table 9). This suggests that the increased *IFNAR2* (but not *IL10RB*) levels observed in the most severe group of COVID-19 patients (Fig. 4a) may reflect the increased likelihood of SARS-CoV-2 viremia in those patients^17^.

The effect of *IL10RB* and *IFNAR2* expression levels on SARS-CoV-2 viral load was replicated in A549-ACE2 cells, which constitute a more relevant cellular context for COVID-19 infection. A549-ACE2^23^ is a human alveolar basal epithelial carcinoma cell line (A549) that constitutively expresses ACE2 (the host receptor required for SARS-CoV-2 entry) and is able to support SARS-CoV-2 infection and replication^24^. Specifically, we verified that *IL10RB* expression is positively correlated with SARS-CoV-2 viral load via siRNA-mediated knockdown of *IL10RB* and exogenous *IL10RB* overexpression (Pearson’s r = 0.88, *p* = 8.5 × 10^–4^; Supplementary Fig. 10; Supplementary Table 10). Consistent with the findings above in NGN2 cells, we also found that *IFNAR2* levels are positively correlated with viral load (Pearson’s r = 0.92, *p* = 4.7 × 10^−5^; Supplementary Fig. 10; Supplementary Table 10).

## Discussion

Our multidisciplinary translational genomics framework integrates GReX and perturbagen signature libraries to identify druggable gene targets for COVID-19 (Fig. 1). Transcriptomic imputation^11,25,26^ serves as the genomics backbone of this approach, and it trades off a part of SNP heritability in exchange for GReX^27^ which has translational potential. We first performed TWAS for all publicly available GWASs for different COVID-19 phenotypes from the COVID-19 Host Genetics Initiative (Supplementary Table 2) and decided to base our downstream analyses on the COVID-19-associated hospitalization vs. population phenotype. We would have preferred to use a GWAS that compares severe, or hospitalized, COVID-19 cases against milder cases (infected controls), but these studies are not sufficiently powered (Supplementary Table 2); in retrospect, neither the flagship paper of the COVID-19 Host Genetics Initiative^5^, nor other TWAS-based studies of COVID-19^28,29^, considered the GWASs based on these case/control definitions. Finally, when interpreting TWAS results, we have to take into account that there are differences in the cohort eligibility and sample collection between cohorts, which will be reflected in the TWAS results. For example, in the STARNET cohort, blood was collected from beating heart donors, whereas in GTEx the samples were obtained mostly (but not exclusively) postmortem. Taking into account that *IL10RB* has been shown to be significantly (FDR = 4.55 × 10^−35^) differentially expressed in pre- and postmortem blood samples in GTEx^30^, we should be cautious when, e.g., directly comparing findings for blood tissue between these two cohorts.

For our novel gene target prioritization approach, we integrated the multi-tissue TWAS results with an shRNA signature library^6^ to identify genes whose perturbation can reverse the disease-associated GReX. This shRNA GReX antagonism approach identified *IL10RB* (21q22.11) as the most promising gene target and overcame traditional limitations of GWAS and TWAS analyses by identifying key genes within a gene cluster. Based on existing approaches, *IL10RB* would not have been the top candidate for further investigation. First, the index SNP for COVID-19 susceptibility in 21q22.11, rs13050728^5^, falls within an intronic region of *IFNAR2* (less than 24kbp from *IL10RB*). In addition, integrating genotype-gene expression datasets cannot identify the most likely causal gene in this locus since the index SNP is associated with gene expression changes of both *IFNAR2 and IL10RB*^5^. Similarly, the genes can only be partially prioritized with targeted individual imputation (Fig. 3a; *IFNAR2* is not associated with COVID-19 death but is associated with COVID-19 severity) and cannot be prioritized with TWAS based on summary statistics (Fig. 2a) even when considering splicing^28^ due to co-regulation^31^ (Supplementary Fig. 11). Finally, Mendelian randomization from the VA Million Veteran Program COVID-19 Science Initiative identified both *IFNAR2* (*p* = 9.8 × 10^−11^) and *IL10RB* (*p* = 2.3 × 10^−14^) with good colocalization properties (PP.H4 > 0.8) and suggested a bigger role for *IFNAR2* based on PheWAS and pathway enrichment analysis for peak instrumental variants^32^. In our approach, *IL10RB* was prioritized primarily because its downregulation in cell lines reverses the GReX signature of COVID-19 and, secondarily, because it has a more uniform imputed transcriptional dysregulation across tissues (predominant downregulation; Fig. 2a, Supplementary Fig. 12). On the other hand, *IFNAR2* is expected to be upregulated in some tissues and downregulated in others^28^ (Fig. 2a, Supplementary Fig. 12), e.g., there was a consistent, predicted, downregulation in adipose tissue and an opposing upregulation in muscle tissue (Supplementary Fig. 13). Unfortunately, technological innovations that would allow differential targeting of tissues are not readily available; thus, our gene target prioritization approach inherently penalizes opposing effects on GReX by integrating multiple tissues (Supplementary Fig. 5). Our study confirms that *IFNAR2* is important for COVID-19 susceptibility^29,32^; however, we prioritized *IL10RB* over *IFNAR2* as a suitable gene target for novel therapeutic development. To our knowledge, there is one other data-driven study that also nominated *IL10RB* from this locus by integrating information derived from single-cell/single-nucleus expression profiling of COVID-19 and healthy tissues (lung, ear, liver, and kidney)^22^.

Toward validating *IL10RB* as a suitable molecular target, we established a direct association between increased *IL10RB* GReX (Fig. 3a) and expression (Fig. 4a) with worse COVID-19 clinical outcomes and death. Importantly, these results provide external validity for our findings beyond individuals of European ancestry (EA)^33^ by performing ancestry-specific analysis for GReX (Fig. 3a) and leveraging a diverse patient cohort for expression profiling (Fig. 4a). Since available multi-tissue reference training datasets comprise mostly EA subjects, there are limitations when performing non-EA GReX association analyses; e.g., differential coverage of SNP predictors across different ancestry groups (Supplementary Table 11). Overall, EA-derived imputation models perform better in similar EA target populations^33^, and a decrease in relative power is observed when performing trans-ethnic association studies where gene-trait associations reach significance only in EA-specific and not the trans-ethic analysis^34^. However, despite these limitations, we did observe good concordance among the ancestry groups in our study (Fig. 3a). Male sex is a strong risk factor for COVID-19; while sex-specific analyses were not performed due to power considerations, both of these analyses were adjusted for sex. Finally, isogenic manipulation of *IL10RB* in a model cell system for SARS-CoV-2 using NGN2 cells^19^ revealed that inducing *IL10RB* expression led to priming of SARS-CoV-2 pathways (Fig. 4d) and increased SARS-CoV-2 viral load upon infection (Fig. 4c) which is associated with worse COVID-19 outcomes^17^. A limitation of this part of the study is the model cell system that we utilized (NGN2 cells); however, this was the only cell system available to us during the challenging times of the pandemic with the capability to perform isogenic overexpression. NGN2 cells have transcriptional regulatory pathways that may differ from e.g., innate immune cells; however, we found that SARS-CoV-2 infection in this cell system mimicked the transcriptional signatures corresponding to SARS-CoV-2 infection of a diverse range of cell types^21^ (Fig. 4d; Supplementary Fig. 9) and identified SARS-CoV-2-relevant pathway dysregulation. Finally, the effect of *IL10RB* and *IFNAR2* expression on SARS-CoV-2 viral load was replicated in a lung alveolar cell line which is a more relevant model system for COVID-19^24^.

It was recently shown that *IL10RB* is significantly upregulated (z-score = 4.16) in ciliated cells in COVID-19 compared with healthy lung^22^. IL10RB serves as a receptor for members of the extended IL-10 family of cytokines (IL10RA_2_IL10RB_2_ heterotetramer for IL-10; IL10RB heterodimers with IL22RA1, IL20RA, and IFNLR1 for IL-22, IL-26 and IFNL1/IFNL2/IFNL3 respectively) which emerged before the adaptive immune response and are essential in modulating host defense mechanisms, especially in epithelial cells, to limit the damage caused by viral and bacterial infections^35^. This family of ligands has diverse and often contradicting roles in host response with an undetermined extent of functional crosstalk between them^36^; thus, further molecular dissection will be required to identify the causal signaling pathway(s) of IL10RB in COVID-19 susceptibility. IL-10 was found to be an important mediator of enhanced susceptibility to respiratory postviral bacterial superinfections in a mouse model^12^. IL-22 promotes antibacterial activity^37^ and enhances tissue regeneration and wound healing^38^. IL-26 is poorly understood. Finally, IFN-λs(IL-28/IL29) are induced by a viral infection and show antiviral activity^39,40^; unsurprisingly, in a COVID-19 mouse model, administration of IFN-λ1a led to diminished SARS-CoV-2 replication^41^. However, the participation of IFN-λs in damaging pro-inflammatory responses remains to be evaluated since recent mouse model studies showed that: (1) viral RNA-induced IFN-λ production causes direct disruption of the lung epithelial barrier and increases susceptibility to bacterial superinfections^13^, and (2) IFN-λ signaling aggravates viral infection by impairing lung epithelial regeneration^42^. Clinical outcomes from pegylated IFN-λ1a clinical trials against COVID-19 will provide evidence about the desired modulation direction of this pathway in COVID-19 treatment (phase II clinical trials: NCT04343976, NCT04354259, NCT04534673, and NCT04344600). Possible next steps include the evaluation of readily available IL-10 neutralizing antibodies (e.g., BT063, Biotest; NCT02554019) and IL-22 neutralizing antibodies (e.g., ILV-094/095, Pfizer). While the molecular dissection of this COVID-19 susceptibility pathway is important, transiently downregulating *IL10RB* with RNA interference^43^ may be sufficient if a lung targeting approach is developed.

## Methods

### Transcriptome-wide association study

#### Transcriptomic imputation model construction

Transcriptomic imputation models were constructed as previously described^11^ for peripheral tissues of the GTEx v8^7^ (excluding brain and tissues with *n* ≤ 73) and STARNET^8^ cohorts (Supplementary Table 1; Fig. 1a). The genetic datasets of the GTEx and STARNET cohorts were uniformly processed for quality control (QC) steps before genotype imputation. We restricted our analysis to samples with European ancestry as previously described^11^. Genotypes were imputed using the University of Michigan server^44^ with the Haplotype Reference Consortium (HRC) reference panel^45^. Gene expression information was derived from RNA-seq gene-level counts, which were adjusted for known and hidden confounds, followed by quantile normalization. For GTEx, we used publicly available, quality-controlled gene expression datasets from the GTEx consortium (http://www.gtexportal.org/). RNA-seq data for STARNET were obtained in the form of residualized gene counts from a previously published study^8^. For the construction of the transcriptomic imputation models, we used elastic net-based methods; when epigenetic annotation information^46^ was available for a given tissue, we employed the EpiXcan^11^ method to maximize power; when not available, we used the PrediXcan^25^ method.

#### COVID-19 phenotypes GWAS summary statistics

Summary statistics for all 7 COVID-19 phenotypes (A1, A2, B1, B2, C1, C2, and D1; Supplementary Table 2) were obtained from the COVID-19 Host Genetics initiative^4^ (Release 4; 2020-10-20; https://www.covid19hg.org/results/r4/).

#### Multi-tissue transcriptome-wide association study (TWAS)

We performed the gene-trait association analysis as previously described^11^. Briefly, we applied the S-PrediXcan method^26^ to integrate the COVID-19 GWAS summary statistics and the transcriptomic imputation models constructed above to obtain gene-level association results.

#### Gene set enrichment analysis for TWAS results

To investigate whether the genes associated with a given trait exhibit enrichment for biological pathways, we used gene sets from MsigDB 5.1^47^ and filtered out nonprotein coding genes, genes located at MHC, as well as genes whose expression could not be reliably imputed. Statistical significance was evaluated with one-sided Fisher’s exact test and the adjusted p values obtained by the Benjamini–Hochberg method^48^. We also performed separately LD-aware TWAS pathway enrichment analysis with JEPEGMIX2-P^9^ v01.1.0 with SNP and gene set annotations v0.3.0.

### Genetically regulated gene expression (GReX-) based gene targeting approach

The gene targeting approach integrates genetically regulated gene expression (GReX) information (using the TWAS gene-trait-tissue association results) with a perturbagen signature library^6^ (Fig. 1).

#### Perturbagen Library used

We used the LINCS Phase II L1000 dataset (GSE70138) perturbagen reference library^6^; specifically the shRNA signatures (gene expression changes after knocking down a gene). All inferred genes (AIG; *n* = 12,328) were considered. Only “gold” signatures were considered.

#### Imputed transcriptomes used

We only considered GReX from 17 EpiXcan tissue models of the B2 phenotype that had significant TWAS results (FDR adjustment was applied to all COVID-19 phenotypes and tissues; Supplementary Table 1; the steps of the method are also outlined in Supplementary Fig. 5).

#### Signature antagonism of trait GReX

Each signature from the shRNA signature library (e.g., *IL10RB* shRNA treatment for 96 h in MCF7 cells) was assessed and ranked for its ability to reverse the trait-associated imputed transcriptomes using a previously published CDR method^10^.

#### Summarization of the effect of signatures across tissues

Signatures were grouped by peturbagen (shRNA), and we first tested whether the signatures for a specific perturbagen were more likely to be ranked higher or lower (Mann–Whitney U test). Then, we obtained a perturbagen-specific GReX antagonism pseudo-z-score which is defined as the negative Hodges-Lehmann estimator (of the median difference between that specific shRNA vs. the other shRNAs) divided by the standard deviation of the ranks of the shRNAs as follows: ${\mathrm{pseudo}}\;z_{GReX\;{\mathrm{antagonism}}} = - \frac{{{\mathrm{Hodges}} - {\mathrm{Lehmann}}\;{\mathrm{estimator}}_{{\mathrm{perturbagen}}}}}{{{\mathrm{SD}}\;{\mathrm{average}}\;{\mathrm{ranks}}\;{\mathrm{of}}\;{\mathrm{all}}\;{\mathrm{perturbagens}}}}$. A positive pseudo-z-score is interpreted as a potential therapeutic candidate, whereas a negative pseudo-z-score would suggest that the shRNA is not antagonizing the imputed transcriptome and is thus likely to exacerbate the phenotype. FDR is estimated with the Benjamini–Hochberg procedure^48^. All shRNAs were considered.

#### Gene prioritization approach

For prioritization, we estimated, for each gene, the *p*-value corresponding to the joint statistic of the two approaches $\left( {z_{{\mathrm{combined}}} = \underline {z_{TWAS}} + {\mathrm{pseudo}}\;z_{GReX\;{\mathrm{antagonism}}}} \right)$.

### IL10RB and IFNAR2 GReX association with COVID-19 severity and other phenotypes in the Million Veteran Program

#### Cohort

Within the broader cohort of the Million Veteran Program^14^, for the COVID-19 severity analysis we used all COVID-19-positive individuals as of March 11, 2021 (n_EUR_ = 14,262, n_AFR_ = 5828, n_HIS_ = 2870, n_ASN_ = 266; EUR: European; AFR: African; HIS: Hispanic; ASN: Asian) from MVP release 4. For the phenome-wide association study (PheWAS), we used individuals of European Ancestry (*n* = 296,407) from MVP release 3. Ancestries were defined by the HARE method^49^. Genotypes used for imputation were filtered by Minor Allele Frequency (>0.01), Variant level Missingness (<0.02), as well as imputation R^2^ (>0.9). We considered the MVP severity cohort an independent cohort from the GWAS since less than 7% (1519) of its participants were included in the COVID-19-related hospitalization GWAS (“B2_ALL_eur_leave_23andme”; release 4), comprising less than 7% and 0.2% of the GWAS’s cases and total individuals, respectively. For the individual imputation, we used the EpiXcan tissue model of blood from the STARNET cohort for the following reasons: (1) as tissue, blood is relevant (immune cells) and accessible—allowing for testing and validation, and (2) as an imputation model, the blood (STARNET) is the most powerful model (identifying the most FDR-significant gene-trait associations; Supplementary Fig. 4), allows the concurrent study of both *IL10RB* and *IFNAR2* (Fig. 2a), and is based on collection from beating heart donors (in contrast to the GTEx model which is based mostly on postmortem blood). These analyses were conducted under the protocol “VA Million Veteran Program COVID-19 Science Initiative”, which was approved by the Veterans Affairs Central Institutional Review Board and by the Research & Development Committee at the James J. Peters VA Medical Center.

#### Phenotypes

There are four COVID-19 severity levels: mild, moderate, severe, and death (see Supplementary Table 12 for more information on the phenotypic definition and counts).

#### Transcriptomic imputation

GReX for blood *IL10RB* and *IFNAR2* was calculated with the EpiXcan^11^ Blood (STARNET) transcriptomic imputation model (Supplementary Table 1). For TWAS, we only considered SNPs with imputation R^2^ ≥ 0.3. Ancestry-specific principal component analysis was performed using the EIGENSOFT^50^ v6 software as previously described^51^.

#### GReX association with COVID-19 severity

Associations of GReX and COVID-19 severity (Supplementary Table 12) were independently performed in each ancestry group. All associations were performed on scaled GReX with the following covariates: Elixhauser comorbidity index^15^ for 2 years, sex, age, age squared (age^2^), and top 10 ancestry-specific principal components. To estimate the effect of GReX on COVID-19-related death, we used a logistic regression analysis (binomial distribution) where death was defined as “1”, while mild, moderate, and severe cases were defined as “0”. To estimate the effect of GReX on COVID-19-related outcome severity, we performed an ordinal logistic regression where COVID-19 severity was ordered as follows: mild, moderate, severe, and death. The ancestry-specific associations were meta-analyzed with a fixed-effect model using the inverse-variance method to estimate the effect of IL10RB and IFNAR2 GReX in the total population.

#### GReX PheWAS

Phecodes^52^ assigned to clinical encounters up to 2018 (predating the COVID-19 pandemic) were grouped into categories using Phecode Map v1.2 with manual curation for some uncategorized phecodes (as provided in Supplementary Data 10). All phecodes with at least one count in more than 25 individuals in the cohort were considered for further analysis. Association testing was performed between scaled GReX and counts of each phecode with a negative binomial distribution—this is the appropriate distribution to capture the full range of phecode counts since variance was higher than the mean phecode count in 99.95% of the phecodes evaluated (1840/1841; the only exception was “Other disorders of purine and pyrimidine metabolism”). The following covariates were used: total number of phecodes per individual, length of the record, sex, age, and top 10 ancestry-specific principal components. Phecodes with nonconvergent regression models were dropped. Significance was tested at the 0.05 false discovery rate (FDR) level.

### Gene expression profiling and EHR-based phenotyping in the Mount Sinai COVID-19 Biobank

Bio-specimens for this analysis were obtained from 568 individuals^16^; some of whom (*n* = 392) contributed more than one bio-specimen. The complete biobank dataset and analyses will be presented in Thompson et al.^53^.

#### RNA-seq gene expression profiling

RNA was extracted from whole blood samples and used to prepare RNA-seq libraries which were quality controlled and sequenced, as previously described^53,54^. In addition, we confirmed that no samples were mislabeled. RNA-seq reads were processed, quality controlled, and aligned to a reference genome as previously described^53,54^. After removing lowly expressed genes (keeping genes with counts per million >1 in at least half of the number of control subjects in the cohort), we normalized the raw count data of the 21,194 remaining genes using voom^55^ with dream^56^ weights from the variancePartition R package^57^. Samples that failed to pass all quality controls were removed from further analyses. Principal component analysis was performed using the prcomp R function to explore covariate effect on gene expression variance genome-wide. Batch effect was calculated on a per gene basis using technical replicates sequenced in all batches, as previously described^53^. The following additional covariates were included in the model: Subject ID, number of days since first blood sample, RNA Library Prep Plate, Sex, DV200 Percent, and PICARD metrics PCT_R2_TRANSCRIPT_STRAND_READS, PCT_INTRONIC_BASES, WIDTH_OF_95_PERCENT, MEDIAN_5PRIME_BIAS, MEDIAN_3PRIME_BIAS^53^. Cell type proportions were calculated with CIBERSORTx^58^ using the LM22 reference^53,59^. COVID-19 severity-associated cell types are identified as having nonzero coefficients in a linear mixed model lasso procedure (R package glmmlasso) predicting COVID-19 severity^53^. Finally, we used dream^56^ (a precision-weighted linear mixed model to consider repeated measures) for differential gene expression analysis while accounting for covariates identified by variancePartition^57^, above, as well as the proportions of COVID-19 severity associated with cell types identified above. A total of 6 DE signatures were generated (Severe end-organ damage Vs. Severe; Severe end-organ damage Vs. Moderate; Severe end-organ damage Vs. Control; Severe Vs. Moderate; Severe Vs. Control; Moderate Vs. Control). Multiple testing was controlled separately for each DE comparison accounting for the 21,114 genes tested using the false discovery rate (FDR) estimation method of Benjamini–Hochberg^48^.

#### COVID-19 severity scale

Phenotypic information was obtained by the EMR of the Mount Sinai Health System, which is reviewed by a screening team that includes practicing physicians. Each bio-specimen was associated, when possible—given the information in the EMR—with a COVID-19 severity measurement that corresponded to the time of collection. There are four levels of severity^60^: controls, moderate, severe, and severe end-organ damage, summarized in Supplementary Table 13.

#### Ethics statement

This study was approved by the Human Research Protection Program at the Icahn School of Medicine at Mount Sinai (STUDY-20-00341). All patients admitted to the Mount Sinai Health System were made aware of the research study by a notice included in their hospital intake packet. The notice outlined details of the specimen collection and planned research. Flyers announcing the study were also posted in the hospital, and a video was run on the in-room hospital video channel. Given the monumental hurdles of consenting sick and infectious patients in isolation rooms, the Human Research Protection Program allowed for sample collection, which occurred at the time of clinical collection and included at most an extra 5–10 cc of blood prior to obtaining research consent. Limited existing clinical data obtained from the medical record was collected and associated with the samples. As the research laboratory processing needed to begin proximal to sample collection, a portion of the data was generated prior to obtaining informed consent. During or after hospitalization, research participants and/or their legally authorized representative provided consent to the research study, including genetic profiling for research and data sharing on an individual level. In those circumstances where consent could not be obtained (13.8% of subjects, 0% of subjects who completed the post-discharge checklist), data already generated could continue to be used for analysis purposes only when not doing so would have compromised the scientific integrity of the work. The data were not identifiable to the researchers doing the analyses.

### Manipulation of *IL10RB* and *IFNAR2* expression in NGN2 cells and their effect on SARS-CoV-2 viral load and transcriptional profiles

#### Overview

NGN2-glutamatergic neurons were derived from hiPSC-NPCs of donor NSB2607^61^ as previously described^62^. gRNAs were designed using the CRISPR-ERA (http://crispr-era.stanford.edu) web tool and cloned into a lentiviral transfer vector using Gibson assembly^18,62^; shRNAs were ordered as glycerol stocks from Sigma. Wild type or dCas9 expressing NPCs were infected with rtTA and NGN2 lentiviruses, as well as desired shRNA or CRISPRa lentiviruses, and differentiated for 7 days before SARS-CoV-2 infection with a multiplicity of infection (MOI) of 0.1 or mock infection for 24 h. After the completion of the experiment, RNA was isolated, quality metrics were obtained, and 200 ng of RNA was processed through a total RNA library prep using the KAPA RNA Hyper Prep Kit + RiboErase HMR kit (Roche, cat no: 8098140702) following the manufacturer’s instructions. The resulting libraries were sequenced on the NovaSeq 6000 S4 flow cell (Illumina), obtaining 2 × 150 bp reads at 60 M reads per sample. An additional 20 ng of RNA was run on a SARS-CoV-2 targeted primer panel using AmpliSeq Library Plus and cDNA Synthesis for Illumina kits (Illumina, Cat no: 20019103 and 20022654). All samples were normalized, pooled, and run on the NovaSeq 6000 S4 in a 2 × 150 run targeting 750 K reads per sample. The STAR aligner v2.5.2a^63^ was used to align reads to the GRCh38 genome (canonical chromosomes only) and Gencode v25 annotation. The module featureCounts^64^ from the Subread package v1.4.3-p1^65^ was used to quantify genes. RSeQC v2.6.1^66^ and Picard v1.77^67^ were used to generate QC metrics. Differential expression analysis was performed with limma^68^ using the first two components of multidimensional scaling and RIN as covariates. Competitive gene set testing using sets from Gene Ontology^69^ and the COVID-19 Drug and Gene Set Library^21^ was performed with camera^70^. SARS-CoV-2 quantification was performed by taxonomically classifying short-read data with taxMaps^71^. The AmpliSeq approach confirmed the presence or absence of the virus in our samples. A more detailed version of this section can be found in the Supplementary Methods section.

#### hiPSC-NPC culture and donor

hiPSC-NPCs of line NSB2607 (male, 15 years old, European descent)^61^ were cultured in hNPC media (DMEM/F12 (Life Technologies #10565), 1× N-2 (Life Technologies #17502-048), 1× B-27-RA (Life Technologies #12587-010), 20 ng/mL FGF2 (Life Technologies)) on Matrigel (Corning, #354230). hiPSC-NPCs at the full confluence (1–1.5 × 10^7^ cells/well of a six-well plate) were dissociated with Accutase (Innovative Cell Technologies) for 5 min, spun down (5 min × 1000 × *g*), resuspended, and seeded onto Matrigel-coated plates at 3–5 × 10^6^ cells/well. Media was replaced every 24 h for 4 to 7 days until the next passage.

#### SARS-CoV-2 virus propagation and infections

SARS-related coronavirus 2 (SARS-CoV-2), isolate USA-WA1/2020 (NR-52281) was deposited by the Center for Disease Control and Prevention and obtained through BEI Resources, NIAID, NIH. SARS-CoV-2 was propagated in Vero E6 cells in DMEM supplemented with 2% FBS, 4.5 g/L D-glucose, 4 mM L-glutamine, 10 mM Non-Essential Amino Acids, 1 mM Sodium Pyruvate and 10 mM HEPES. Virus stock was filtered by centrifugation using Amicon Ultra-15 Centrifugal filter unit (Sigma, Cat # UFC910096) and resuspended in viral propagation media. All infections were performed with either passage 3 or 4 SARS-CoV-2. Infectious titers of SARS-CoV-2 were determined by plaque assay in Vero E6 cells in Minimum Essential Media supplemented with 4mM L-glutamine, 0.2% BSA, 10 mM HEPES and 0.12% NaHCO3, and 0.7% Oxoid agar (Cat #OXLP0028B). All SARS-CoV-2 infections were performed in the CDC/USDA-approved BSL-3 facility of the Global Health and Emerging Pathogens Institute at the Icahn School of Medicine at Mount Sinai in accordance with institutional biosafety requirements.

#### gRNA design and cloning and shRNAs

gRNAs were designed using the CRISPR-ERA (http://crispr-era.stanford.edu) web tool. gRNAs were selected based on their specific locations at decreasing distances from the TSS as well as their lack of predicted off-targets and E scores (http://crispr-era.stanford.edu). For lentiviral cloning: synthesized oligonucleotides were phospho-annealed (37 °C for 30 min, 95 °C for 5 min, ramped-down to 25 °C at 5 °C per min), diluted 1:100, ligated into BsmB1-digested lentiGuide-Hygro-mTagBFP2 (addgene Plasmid #99374) and transformed into NEB10-beta E. coli, according to manufacturer’s instructions (NEB # C3019H). shRNAs were ordered as glycerol stocks from Sigma (IL10RB # SHCLNG-NM_000628; IFNAR2 # SHCLNG-NM_000874). Gibson Assembly of Vectors: Unless specified, all cloning reagents were from NEB, and plasmid backbones were from Addgene (https://www.addgene.org/). Primers were synthesized by Thermo Fisher Scientific. All fragments were assembled using NEBuilder HiFi DNA Assembly Master Mix (NEB, no. E2621X). All assemblies were transformed into either DH5a Extreme Efficiency Competent Cells (Allele Biotechnology, no. ABP-CE-CC02050) or Stbl3 Chemically Competent E. coli (Thermo Fisher Scientific, no. C737303). Positive clones were confirmed by restriction digest and Sanger sequencing (GENEWIZ). The following vectors have been deposited at Addgene: lenti-EF1a- dCas9-VP64-Puro, lenti-EF1a-dCas9-VPR-Puro, lenti-EF1a-dCas9-KRAB-Puro, lentiGuide-Hygro-mTagBFP2, lentiGuide-Hygro-eGFP, lentiGuide-Hygro-dTomato, lentiGuide-Hygro-iRFP670, and pLV-TetO-hNGN2-Neo. lentiGuide-dTomato and lentiGuide-mTagBFP2-Hygro lentiGuide-Puro (Addgene, no. 52963) were digested with Mlu1 and BsiWI. dTomato was amplified from AAV-hSyn1-GCaMP6f-P2A-NLS-dTomato (Addgene, no.51085). HygroR sequence was amplified from lentiMS2-P65-HSF1_Hygro (Addgene, no. 61426). mTagBFP2 was amplified from pBAD-mTagBFP2 (Addgene, no. 3463). The P2A self-cleaving peptide sequence was amplified using a reverse primer of HygroR and a forward primer of mTagBFP2. All sequences are provided in Supplementary Table 14.

#### Lentiviral dCas9 effectors

To engineer a lentiviral transfer vector that expresses dCas9: VP64-T2A-Puro (EF1a-NLS-dCas9(N863)-VP64-T2A-Puro-WPRE), dCas9:VP64-T2A-Blast (EF1a-NLS-dCas9(N863)-VP64-T2A-Blast-WPRE) (Addgene, no. 61,425) was digested with BsrGI and EcoRI. T2A-PuroR was amplified from pLV-TetO-hNGN2-P2A-eGFP-T2A-Puro (Addgene, no. 79823). Fragments were then assembled using NEBuilder HiFi DNA Assembly Master Mix (NEB, no. E2621). To engineer a lentiviral transfer vector that expresses dCas9:KRAB-Puro (EF1a-NLS-dCas9(N863)-KRAB-T2A-Puro-WPRE), dCas9:VP64-T2A-Blast (EF1a-NLS-dCas9(N863)-VP64-T2A-Blast-WPRE) (Addgene, no.61425) was first digested with BamHI and BsrGI. KRAB was then amplified from pHAGE-TRE-dCas9:KRAB (Addgene, no. 50917). Fragments were assembled using NEBuilder HiFi DNA Assembly Master Mix. dCas9:KRAB-Blast was digested with BsrGI and EcoRI, and T2A-PuroR was amplified from pLV-TetO-hNGN2- P2A-eGFP-T2A-Puro (Addgene, no. 79823). Fragments were then assembled using NEBuilder HiFi DNA Assembly Master Mix. To engineer a lentiviral transfer vector that expresses dCas9:VPR-Puro (EF1a- NLS-dCas9(D10A, D839A, H840A, and N863A)-VPR-T2A-Puro- WPRE), dCas9:VPR was first amplified from SP-dCas9-VPR (Addgene, no. 63798), and T2A-PuroR was amplified from pLV-TetO-hNGN2- P2A-eGFP-T2A-Puro (Addgene, no. 79823). dCas9:KRAB-T2A-Puro was digested with BsiWI and EcoRI. Fragments were then assembled using NEBuilder HiFi DNA Assembly Master Mix.

#### NGN2-glutamatergic neuron induction of shRNA and CRISPRa treated neurons^18,62^

On day -1 NPCs were dissociated with Accutase Cell Detachment Solution for 5 min at 37 °C, counted, and seeded at a density of 5 × 10^5^ cells/well on Matrigel-coated 24-well plates in hNPC media (DMEM/F12 (Life Technologies #10565), 1× N-2 (Life Technologies #17502-048), 1× B-27-RA, 20 ng/mL FGF2 (Life Technologies)) on Matrigel (Corning, #354230). On day 0, cells were transduced with rtTA and NGN2 lentiviruses as well as desired shRNA or CRISPRa viruses in NPC media containing 10 μM Thiazovivin and spinfected (centrifuged for 1 h at 1000 × *g*). On day 1, media was replaced, and doxycycline was added with 1 ug/mL working concentration. On day 2, transduced hNPCs were treated with corresponding antibiotics to the lentiviruses (1 μg/mL puromycin for shRNA, 1 mg/mL G-418 for NGN2-Neo). On day 4, the medium was switched to Brainphys neuron medium containing 1 μg/mL dox. The medium was replaced every second day until SARS-CoV-2 (MOI of 0.1) or mock infection on day 7. The samples were harvested in Trizol (Invitrogen, Cat #15596026) 24 h later. RNA was isolated by phenol/chloroform extraction prior to purification using the RNeasy Mini Kit (Qiagen, Cat # 74106).

#### Sequencing platform

RNA samples were submitted to the New York Genome Center and, following an initial quality check, were normalized onto two different 96-well plates for a total RNA with RiboErase assay and a SARS-CoV-2 targeted assay. For the total RNA assay, 200 ng of RNA were normalized into a plate to be run through the KAPA RNA Hyper Prep Kit + RiboErase HMR (Roche, cat no: 8098140702). This total RNA prep followed the manufacturer’s protocol with minor adjustments for automation on the PerkinElmer sciclone. Briefly, the RNA first goes through an oligo hybridization and rRNA depletion, followed by 1st and 2nd strand synthesis. The cDNA was then adenylated, and unique dual indexed adapters ligated onto the ends. Finally, samples were cleaned up, enriched, and purified. The final library was quantified by picogreen and ran on a fragment analyzer to determine the final library size. Samples were normalized, pooled and run on a NovaSeq 6000 S4 in a 2 × 150 bp run format, targeting 60 M reads per sample. For the SARS-CoV-2 targeted assay, we used the AmpliSeq Library Plus and cDNA Synthesis for Illumina kits (Illumina, Cat no: 20019103 & 20022654). Briefly, 20 ng of RNA were reverse transcribed, the cDNA targets were then amplified with the Illumina SARS-CoV-2 research panel (Illumina, 20020496). The amplicons were partially digested, and AmpliSeq CD Indexes were ligated onto the amplicons. The library was then cleaned up and amplified. After amplification, there was a final clean-up, and the libraries were quantified, pooled, and run on a NovaSeq 6000 S4, obtaining 2 × 150 bp reads.

#### SARS-CoV-2 quantification

Short-read data were taxonomically classified using taxMaps^71^. As part of the taxMaps pipeline, reads were processed prior to mapping. Adapter sequences and low-quality (Q < 20) bases were trimmed out, and low complexity reads discarded. The remaining reads were then concurrently mapped against (1) the phiX174 reference genome (NC_001422.1); (2) the SARS-CoV-2 reference genome (NC_045512.2); and (3) a combined index encompassing the entire NCBI’s nt database, RefSeq archaeal, bacterial, fungal, protozoan and viral genomes, as well as a selection of RefSeq model organism genomes, including the human GRCh38 reference^72^, to produce the final classification. Given that some human sequences of ancestral origin (that constitute variation between individuals) are absent from the GRCh38 reference, a small percentage of human reads usually maps to other primate genomes and, consequently, was classified as such. To obtain more accurate estimates of the human content in these samples, all reads classified as “primate” were considered of human origin and reclassified accordingly. SARS-CoV-2 viral load was determined as the number of SARS-CoV-2 reads over the host (human) reads.

#### Competitive gene set testing

Competitive gene set testing using sets from Gene Ontology^69^ and the COVID-19 Drug and Gene Set Library^21^ was performed with camera^70^. First, we performed differential expression analysis with limma^68^ using the first two components of multidimensional scaling and RIN as covariates to identify the signature of SARS-CoV-2 infection in our cells while adjusting for other treatments. We then performed competitive gene set enrichment analysis for all gene ontology and betacoronavirus gene sets (*n* = 18,553). For gene ontology datasets, we kept all significantly enriched gene sets (FDR < 0.05) and kept those with a Jaccard index less than 0.2. For the betacoronavirus gene sets, we kept all the gene sets and filtered them based on a Jaccard index of 0.2. The combined SARS-CoV-2 gene set collection with the two datasets above (significantly pruned gene ontology and all pruned betacoronavirus) was used for all the following competitive gene set testing except as otherwise indicated (*n* = 296). Thus, in Fig. 4d, enrichment analysis is run across the whole exploratory dataset (*n* = 18,553) for SARS-CoV-2 infection (first row), whereas for all other conditions, we are only exploring the combined SARS-CoV-2 gene set collection (*n* = 296).

### Manipulation of *IL10RB* and *IFNAR2* expression in A549-ACE2 alveolar cells and their effect on SARS-CoV-2 viral load

#### Overview

ACE2-expressing A549 cells (A549-ACE2), a gift from Brad Rosenberg^23^, were either transfected with siRNA, or transduced with the TetOne inducible system prior to infection with SARS-CoV-2. For knockdown, A549-ACE2 cells were transfected with pooled siRNAs (Dharmacon) 48 h prior to SARS-CoV-2 infection. For overexpression, A549-ACE2 cells were transduced using the TetOne Inducible Expression System (Takara Bio) with lentivirus-containing TetOne-eGFP, -IL10RB, or -IFNAR2 for 48 h followed by puromycin selection. To induce gene expression, A549-ACE2 TetOne-eGFP, -IL10RB, or -IFNAR2 cells were treated with either 0 ng/mL or 100 ng/mL doxycycline for 24 h before SARS-CoV-2 infection. Cells were either mock-infected or infected with media containing SARS-CoV-2 for a multiplicity of infection (MOI) of 0.02. The cells were incubated for 48 h, and then the cells were harvested, and the virus was inactivated by Trizol (Invitrogen) or RIPA lysis buffer for safe removal from the BSL-3 facility. Lysates were stored at −80 °C until further analysis. RNA was isolated from Trizol, cleaned using the Qiagen RNeasy Mini Kit (Cat # 74106), and quantified by QuBit. A starting input of 500 ng was used to prepare cDNA via the High-Capacity cDNA Reverse Transcription Kit (Applied Biosystems, Cat # 4368813). Real-time qPCR was performed using TaqMan probes (Thermo Fisher Scientific) for the SARS-CoV-2 S (vi07918636_s1), *IL10RB* (hs00175123_m1), *IFNAR2* (hs01022059_m1) and control gene, *GAPDH* (hs02786624_g1). qPCR reactions were performed on the Applied Biosystems QuantStudio 5 and analyzed using the -ΔΔCt method for fold change expression to validate genetic manipulation and quantify SARS-CoV-2 infection. A more detailed version of this section can be found in the Supplementary Methods section.

#### Plasmids

The pLVX.TetOne-2xstrept-eGFP plasmid was a gift from Nevan Krogan^73^. pLVX.TetOne-IL10RB and pLVX.TetOne-IFNAR2 where cloned using the parental pLVX-TetOne vector (Takara Bio, Inc.) and cDNA encoding human IL10RB or human IFNAR2 (IDT) with the InFusion HD Cloning Kit (Takara Bio, Inc.). Clones were sequence-verified by sanger sequencing (Psomagen). Sequences are provided in Supplementary Table 14.

#### Cells

HEK 293 T cells, a human kidney epithelial cell line (HEK 293 T/17, ATCC®, CRL-11268); and Vero E6 cells (Vero 76, clone E6, Vero E6, ATCC® CRL-1586; for SARS-CoV-2 propagation), an African Green Monkey kidney epithelial cell line, were authenticated by ATCC. A monoclonal ACE2-expressing A549 cell line (A549-ACE2) was a gift from Brad Rosenberg^23^. All cell lines were cultured under humidified, 5% CO_2_, 37 °C conditions in complete DMEM (10% v/v fetal bovine serum (FBS, Thermo Fisher Scientific) and 100 I.U. penicillin and 100 µg/mL streptomycin (Pen/Strept, Corning) in Dulbecco’s Modified Eagle Medium (DMEM, Corning)). Cells were confirmed negative for mycobacteria monthly (Lonza).

#### Viruses

SARS-related coronavirus 2 (SARS-CoV-2) isolate USA-WA1/2020 (NR-52281) was obtained from BEI Resources, NIAID, NIH. Virus stocks were grown, processed, and titrated as previously described^74^. The supernatant was collected 30 h postinfection and concentrated through a 100 kDa centrifugal filter unit (Amicon). All work with live SARS-CoV-2 was performed in the CDC/USDA-approved biosafety level 3 (BSL-3) facility of the NYU Grossman School of Medicine in accordance with institutional guidelines.

#### Generation and induction of A549-ACE2 TetOne cell lines

In a 6-well format, for each well, 1.5 × 10^6^ HEK 293 T cells were transfected with a mixture of 0.75 µg pLVX.TetOne-strept-eGFP, pLVX.TetOne-IL10RB, or pLVX.TetOne-IFNAR2; 0.75 µg gag/pol lentivirus packaging plasmid (Takara Bio Inc.), and 0.12 µg vesicular stomatitis virus G plasmid ((Takara Bio Inc.) in Opti-MEM (Corning) and Lipofectamine 2000 transfection reagent (Invitrogen) at a µg of DNA: µL of reagent ratio of 1:3. Twenty-four hours post-transfection, media was replaced with complete DMEM. Forty-eight hours post-transfection, cell supernatants were filtered through a 0.22 µm syringe filter. 4 × 10^6^ A549-ACE2 cells in a six-well format were then transduced per well, with 0.7 mL filtered lentivirus-containing supernatant diluted to a total of 2 mL with complete DMEM supplemented with 10 µg/mL polybrene (Corning). Forty-eight hours post-transduction, the supernatant was removed, and fresh complete DMEM containing 2.5 µg/mL puromycin was added to the transduced cells. Forty-eight hours post-puromycin treatment, cells were expanded for use in experiments. Cells were then maintained in 2.5 µg/mL puromycin when in culture. To induce gene expression prior to infection, 2 × 10^5^ A549-ACE2 TetOne-eGFP, -IL10RB, or -IFNAR2 cells were plated in a 24-well format in complete DMEM containing 0 ng/mL or 100 ng/mL doxycycline. Twenty-four hours post-induction, cells were infected as described below.

#### siRNA transfections

2 × 10^5^ A549-ACE2 cells in a 24-well format were transfected per well with 0.5 µL 10 µM pooled siRNAs (Dharmacon) in 50 µL Opti-MEM with 1.5 µL RNAiMAX (Invitrogen). The transfection mix was incubated at room temperature for 15 min prior to addition to cells. Forty-eight hours post-transfection, cells were infected as described below. Sequences are provided in Supplementary Table 14.

#### SARS-CoV-2 infections

Media was removed from cells and mock-infected with 0.5 mL infection media (DMEM, 2% FBS, P/S) or infected with 0.5 mL infection media containing enough SARS-CoV-2 for a multiplicity of infection (MOI) of 0.02. The cells were then incubated under humidified, 5% CO_2_, 37 °C conditions. Forty-eight hours postinfection, media was removed from cells, and 0.5 mL Trizol (Invitrogen) or 0.15 mL RIPA lysis buffer (50 mM Tris HCL, 150 mM NaCl, 0.5% v/v NP-40, 1% v/v Triton X-100, 0.1% w/v SDS, Pierce protease inhibitors) was added to each well. Cells were then incubated at 4 °C for 15 min. Lysates were then transferred to tubes and removed from the BSL-3 facility. Lysates were stored at −80 °C until they were analyzed.

#### SARS-CoV-2 quantification

RNA was isolated from Trizol (Invitrogen) using the manufacturer-provided protocol. Isolated RNA was further cleaned using the Qiagen RNeasy Mini Kit (Cat # 74106) and supplemented with RNAse inhibitor (Takara, Cat # 2313 A) 5% by volume. RNA was quantified by QuBit, and a starting input of 500 ng was used to prepare cDNA via the High-Capacity cDNA Reverse Transcription Kit (Applied Biosystems, Cat # 4368813). TaqMan probes for the SARS-CoV-2 S protein (vi07918636_s1), IIL10RB (hs00175123_m1), IFNAR2 (hs01022059_m1), and control gene, GAPDH (hs02786624_g1), were acquired through Thermo Fisher (Cat # 4331182). Real-time PCR was performed in triplicate using 2 ng of cDNA per reaction and the Applied Biosystems TaqMan Gene Expression Mix (Cat # 4369016). Reactions were run using the Applied Biosystems QuantStudio 5, and SARS-CoV-2 was quantified using the delta-delta Ct method.

#### Statistical analysis

For pairwise comparisons, unpaired *t*-test was used, as indicated in Supplementary Table 10. For correlation analysis of *IL10RB* and *IFNAR2* levels with SARS-CoV-2 viral load, Pearson correlation analysis was used.

### Reporting summary

Further information on research design is available in the Nature Research Reporting Summary linked to this article.

## Supplementary information

Reporting Summary

Supplementary Information

Supplementary Data 1

Supplementary Data 2

Supplementary Data 3

Supplementary Data 4

Supplementary Data 5

Supplementary Data 6

Supplementary Data 7

Supplementary Data 8

Supplementary Data 9

Supplementary Data 10

## Data Availability

All data associated with this study are present in the main text or the Supplementary Data files. Sequencing data from the in vitro experiments (NGN2 cells) have been uploaded to the NCBI Gene Expression Omnibus (GEO; http://www.ncbi.nlm.nih.gov/geo) database under accession number GSE180622. Our study only does targeted replication in the Mount Sinai COVID-19 Biobank; the complete biobank dataset and analyses will be presented in Thompson et al.^53^.
