# Supplementary material for: A translational genomics approach identifies IL10RB as the top candidate gene target for COVID-19 susceptibility
Source: NPJ Genom Med. 2022 Sep 5;7:52. doi: 10.1038/s41525-022-00324-x (PMC9441828; doi:10.1038/s41525-022-00324-x)
Supplement: Supplementary file 1 — Reporting Summary [file 41525_2022_324_MOESM1_ESM.pdf]

nature portfolio

Corresponding author(s): Georgios Voloudakis and Panos Roussos

Last updated by author(s): May 8, 2022

Reporting Summary

Nature Portfolio wishes to improve the reproducibility of the work that we publish. This form provides structure for consistency and transparency in reporting. For further information on Nature Portfolio policies, see our Editorial Policies and the Editorial Policy Checklist.

Statistics

For all statistical analyses, confirm that the following items are present in the figure legend, table legend, main text, or Methods section.

n/a	Confirmed	
☐	☒	The exact sample size (*n*) for each experimental group/condition, given as a discrete number and unit of measurement
☐	☒	A statement on whether measurements were taken from distinct samples or whether the same sample was measured repeatedly
☐	☒	The statistical test(s) used AND whether they are one- or two-sided<br>*Only common tests should be described solely by name; describe more complex techniques in the Methods section.*
☐	☒	A description of all covariates tested
☐	☒	A description of any assumptions or corrections, such as tests of normality and adjustment for multiple comparisons
☐	☒	A full description of the statistical parameters including central tendency (e.g. means) or other basic estimates (e.g. regression coefficient) AND variation (e.g. standard deviation) or associated estimates of uncertainty (e.g. confidence intervals)
☐	☒	For null hypothesis testing, the test statistic (e.g. *F*, *t*, *r*) with confidence intervals, effect sizes, degrees of freedom and *P* value noted<br>*Give P values as exact values whenever suitable.*
☒	☐	For Bayesian analysis, information on the choice of priors and Markov chain Monte Carlo settings
☒	☐	For hierarchical and complex designs, identification of the appropriate level for tests and full reporting of outcomes
☐	☒	Estimates of effect sizes (e.g. Cohen's *d*, Pearson's *r*), indicating how they were calculated

Our web collection on statistics for biologists contains articles on many of the points above.

Software and code

Policy information about availability of computer code

Data collection	TWAS-based methods: Data were collected through the online portals and/or websites as described in the main text and Supplementary Appendix 1. GWAS summary statistics: https://www.covid19hg.org/results/r4/, STARNET EpiXcan models are available at: https://zhangw17.u.hpc.mssm.edu/epixcan/about.php#dl and https://predictdb.org/post/2019/08/23/epixcan-models-integrated-epigenetics/. GTEx reference datasets: http://www.gtexportal.org/. MSigDB: http://software.broadinstitute.org/gsea/msigdb. REMC: https://egg2.wustl.edu/roadmap/web_portal/. GSE70138-associated files: ftp://ftp.ncbi.nlm.nih.gov/geo/series/GSE70nnn/GSE70138/suppl/GSE70138%5FBroad%5FLINCS%5FLevel5%5FCOMPZ%5Fn118050x12328%5F2017%2D03%2D06%2Egctx%2Egz, ftp://ftp.ncbi.nlm.nih.gov/geo/series/GSE70nnn/GSE70138/suppl/GSE70138%5FBroad%5FLINCS%5Fsig%5Finfo%5F2017%2D03%2D06%2Etxt%2Egz and ftp://ftp.ncbi.nlm.nih.gov/geo/series/GSE70nnn/GSE70138/suppl/GSE70138%5FBroad%5FLINCS%5Fgene%5Finfo%5F2017%2D03%2D06%2Etxt%2Egz. JEPEGMIX2-P SNP and gene set annotations v.0.3.0: https://www.dropbox.com/sh/doxfref8zvempr8/AACBg0rMUJtxN7X26G707SiFa?dl=0<br><br>MVP: We didn't engage in any data collection in this project, this is independently handled by the Million Veteran Program Data Core team. For general information please refer to PMID: 26441289, for genotyping array design and data QC refer to PMID: 32243820<br><br>Mount Sinai COVID-19 Biobank: REDCap 10.0.25, Clarity, Caboodle, Epic<br><br>In vitro (NGN2/A549-ACE2): RNA was quantified using the Invitrogen QuBit 2.0 Fluorometer #Q32866 before performing qPCR for the genes of interest. qPCR was performed using the Applied Biosystem QuantStudio 5 #A28135 to quantify expression levels of COVID-spike protein, GAPDH, IL10RB and IFNAR2.
Data analysis	TWAS-based methods: Michigan Imputation Server (HRC reference panel), R >= 3.6, Python 2.7, PrediXcan v1 (model training), EpiXcan v1 (model training), S-PrediXcan v0.6.5, JEPEGMIX2-P v01.1.0<br><br>MVP: Release 3 QC (plink v2), Release 4 QC (by MVP core data team; PMID: 32243820), R >=3.6., Python 2.7, (function calcVarPart of the variancePartition v1.2.5), PrediXcan v1 (for individual imputation), VGAM (ordinal regression)

Mount Sinai COVID-19 Biobank: R v4, Python v3.7.3, MultiQC, bcl2fastq, STAR v2.7.3a, fastqc v0.11.8, Picard Tools v2.22.3, kallisto v0.46.1, Subread v1.6.3, NGSCheckMate (https://github.com/DarwinAwardWinner/GSCheckMate@45160a34acefa81e123cc2bd395b52937e66e0e2), Bioconductor v3.12, limma, voom, glmmlasso, variancePartition, CIBERSORTx (no version number is provided), glmmLasso v1.5.1

In vitro (NGN2/A549-ACE2): qPCR analysis was conducted using the Applied Biosystems QuantStudio™ Design and Analysis Software v1.5.1 for Comparative CT (delta-delta-CT). qPCR was performed in technical triplicate taking the average CT value for each sample for downstream analysis.

For manuscripts utilizing custom algorithms or software that are central to the research but not yet described in published literature, software must be made available to editors and reviewers. We strongly encourage code deposition in a community repository (e.g. GitHub). See the Nature Portfolio guidelines for submitting code & software for further information.

Data

Policy information about availability of data

All manuscripts must include a data availability statement. This statement should provide the following information, where applicable:

- Accession codes, unique identifiers, or web links for publicly available datasets
- A description of any restrictions on data availability
- For clinical datasets or third party data, please ensure that the statement adheres to our policy

TWAS-based methods: The final generated data will be available with publication. Intermediate data generated during the analysis will be available upon requests to corresponding authors.

MVP: The final summary-level data will be available with publication.

Mount Sinai COVID-19 Biobank: All consented data used will be deposited in public repositories upon publication of flagship paper (preprint DOI: https://doi.org/10.1101/2021.10.04.21264434)

In vitro (NGN2/A549-ACE2)): The in vitro data have been uploaded to the NCBI Gene Expression Omnibus (GEO; http://www.ncbi.nlm.nih.gov/geo) database under accession number GSE180622. Dataset access will be switched from private to public status upon publication (https://www.ncbi.nlm.nih.gov/geo/query/acc.cgi?)acc=GSE180622.

Field-specific reporting

Please select the one below that is the best fit for your research. If you are not sure, read the appropriate sections before making your selection.

☒ Life sciences ☐ Behavioural & social sciences ☐ Ecological, evolutionary & environmental sciences

For a reference copy of the document with all sections, see nature.com/documents/nr-reporting-summary-flat.pdf

Life sciences study design

All studies must disclose on these points even when the disclosure is negative.

Sample size

TWAS-based methods: Only sample size consideration was for the use of transcriptomic imputation models with reference data from n>73 individuals.

MVP: All available individuals were used for the association with COVID-19 phenotypes (MVP release 4;nEUR = 14,262, nAFR = 5,828, nHIS = 2,870, nASN = 266; EUR: European; AFR: African; HIS: Hispanic; ASN: Asian); MVP release 3 was used for PheWAS to reflect the pre-pandemic cohort (nEUR = 296,407).

Mount Sinai COVID-19 Biobank: COVID-19 cases (N = 495), Control subjects (N = 72), Blood samples with RNA-seq (N = 1392), Blood samples with RNA-seq & serology (N = 1301), COVID-19 subjects with checklists (N = 251), Control subjects with checklists (N = 15), COVID-19 subjects with RNA-seq & checklists (N = 182), COVID-19 subjects with RNA-seq & serology & checklists (N = 176)

In vitro (NGN2/A549-ACE2): Experiments were designed to be performed as triplicates for each condition. For the IL10RB over-expression in NGN2 cells we used 4 different gRNAs.

Data exclusions

TWAS-based methods: No data were excluded from the analysis

MVP: For both COVID-19 severity and PheWAS association studies, individuals whose genotypes did not pass the quality control as described in the Methods section. For the PheWAS, individuals of non-European ancestry were excluded since cross-ancestry GReX-based PheWAS approaches were not carried out for simplification based on results from the COVID-19 severity analysis.

Mount Sinai COVID-19 Biobank: All COVID-19 cases had positive SARS-CoV-2 PCR test or serology within 2 weeks of initial sampling. All samples passing quality control were included.

In vitro (NGN2/A549-ACE2): Only data that were excluded were the failed qPCR experiments in the A549-ACE2 arm of the study in which case the n is disclosed (n=2 instead of n=3) and only the appropriate amount of data points is shown in the graph.

Replication

TWAS-based methods/MVP: The MVP COVID-19 section of the paper replicated the findings of the TWAS.

Replication	Mount Sinai COVID-19 Biobank: This part of the analysis itself replicated the predicted findings from TWAS and MVP for IL10RB. Replication within this experimental arm was addressed when analyzing cell type deconvolution by using 4 separate references.<br><br>In vitro (NGN2/A549-ACE2): These experiments were the in vitro validation testing the hypotheses from the TWAS, MVP and Mount Sinai COVID-19 Biobank. The A549-ACE2 experimental arm was a limited replication of the NGN2 experimental arm.
Randomization	TWAS-based methods/MVP: N/A (observational study). Relevant variables were controlled for in all statistical models.<br><br>Mount Sinai COVID-19 Biobank: Thorough randomization was performed for biological variables of interest before assignment to sequencing batches. Relevant variables were controlled for in all linear models.<br><br>In vitro (NGN2/A549-ACE2): N/A
Blinding	TWAS-based methods/MVP: N/A (observational study)<br><br>Mount Sinai COVID-19 Biobank: This was not a randomized control trial; patients were recruited as they presented to the hospital, making blinding unneeded for this study.<br><br>In vitro (NGN2/A549-ACE2): Different experimenters performed the treatment and transcript quantification; however, if they wanted they could deduct treatment groups by the sample labels.

Reporting for specific materials, systems and methods

We require information from authors about some types of materials, experimental systems and methods used in many studies. Here, indicate whether each material, system or method listed is relevant to your study. If you are not sure if a list item applies to your research, read the appropriate section before selecting a response.

Materials & experimental systems

n/a	Involved in the study
☒	☐ Antibodies
☐	☒ Eukaryotic cell lines
☒	☐ Palaeontology and archaeology
☒	☐ Animals and other organisms
☐	☒ Human research participants
☒	☐ Clinical data
☒	☐ Dual use research of concern

Methods

n/a	Involved in the study
☒	☐ ChIP-seq
☒	☐ Flow cytometry
☒	☐ MRI-based neuroimaging

Eukaryotic cell lines

Policy information about cell lines

Cell line source(s)	TWAS-based methods/MVP/Mount Sinai COVID-19 Biobank: N/A<br><br>In vitro (NGN2/A549-ACE2): For all studies including NGN2 neurons, parental hiPSCs lines were generated from human skin biopsies reprogrammed using the Yamanaka factors to create pluripotent stem cells. NGN2 neurons were generated from hiPSCs by overexpressing NGN2 via lentiviral transduction. A549 cell lines were acquired from and authenticated by ATCC. The A549-ACE2 cell line was a gift from the lab of Brad Rosenberg (the A549 parental cell line was authenticated by ATCC).
Authentication	TWAS-based methods/MVP/Mount Sinai COVID-19 Biobank: N/A<br><br>In vitro (NGN2/A549-ACE2): NGN2 neurons were previously authenticated via immunostaining for microtubule-associated protein 2AB (MAP2AB). A549 cell lines were acquired from and authenticated by ATCC. Genetic manipulations were confirmed by RT-qPCR using the TaqMan probes for the SARS-CoV-2 S protein (vi07918636_s1), IL10-RB (hs00175123_m1), IFNAR-2 (hs01022059_m1) and control gene, GAPDH (hs02786624_g1) which were acquired and authenticated by the manufacturer, Thermo Fisher Scientific.
Mycoplasma contamination	TWAS-based methods/MVP/Mount Sinai COVID-19 Biobank: N/A<br><br>In vitro (NGN2/A549-ACE2): All cell lines were confirmed mycoplasma-negative monthly.
Commonly misidentified lines<br>(See ICLAC register)	TWAS-based methods/MVP/Mount Sinai COVID-19 Biobank: N/A<br><br>In vitro (NGN2/A549-ACE2): ICLAC-registered misidentified lines include: HEK 293T, which were only used in this study as a tool to generate lentivirus.

Human research participants

Policy information about studies involving human research participants

Population characteristics	TWAS-based methods: N/A. We didn't engage in any data collection in this project. For info please refer to https://www.covid19hg.org/<br><br>MVP: N/A. We didn't engage in any data collection in this project, this is independently handled by the Million Veteran Program Data Core team. For general information please refer to PMID: 26441289.<br><br>Mount Sinai COVID-19 Biobank: Population characteristics are detailed in Table S5. Subjects ranged in age from 0 to 89 or more years old (mean = 62.25, standard deviation = 17.2). COVID-19 cases were all hospitalized, while SARS-CoV-2 negative controls were approximately half hospitalized and half healthy controls. The majority of subjects had at least one comorbidity. 325 subjects were male and 242 were female.<br><br>In vitro (NGN2/A549-ACE2): N/A (not human subjects research)
Recruitment	TWAS-based methods: N/A. We didn't engage in recruitment in this project. For info please refer to https://www.covid19hg.org/<br><br>MVP: N/A. We didn't engage in recruitment in this project, this is independently handled by the Million Veteran Program Data Core team. For general information please refer to PMID: 26441289.<br><br>Mount Sinai COVID-19 Biobank: COVID-19 cases and hospitalized controls were recruited as the presented in the hospital during the pandemic. Healthy controls were recruited from research personnel working at the hospital during the pandemic. PASC checklists were filled only for subjects who chose to respond, potentially representing a self-selection bias.<br><br>In vitro (NGN2/A549-ACE2): N/A (not human subjects research)
Ethics oversight	TWAS-based methods: N/A. We didn't engage in any data collection in this project. For info please refer to https://www.covid19hg.org/<br><br>MVP: VA Central Institutional Review Board as well as local IRBs at all VA sites recruiting participants.<br><br>Mount Sinai COVID-19 Biobank: Human Research Protection Program at the Icahn School of Medicine at Mount Sinai (STUDY-20-00341)<br><br>In vitro (NGN2/A549-ACE2): N/A (not human subjects research)

Note that full information on the approval of the study protocol must also be provided in the manuscript.
