# Supplementary material for: A translational genomics approach identifies IL10RB as the top candidate gene target for COVID-19 susceptibility
Source: NPJ Genom Med. 2022 Sep 5;7:52. doi: 10.1038/s41525-022-00324-x (PMC9441828; doi:10.1038/s41525-022-00324-x)
Supplement: Supplementary file 2 — Supplementary Information [file 41525_2022_324_MOESM2_ESM.pdf]

Supplementary Information

SUPPLEMENTARY FIGURES 3

Supplementary Fig. 1 3

Supplementary Fig. 2 4

Supplementary Fig. 3 5

Supplementary Fig. 4 6

Supplementary Fig. 5 7

Supplementary Fig. 6 9

Supplementary Fig. 7 10

Supplementary Fig. 8 11

Supplementary Fig. 9 12

Supplementary Fig. 10 13

Supplementary Fig. 11 14

Supplementary Fig. 12 15

Supplementary Fig. 13 16

SUPPLEMENTARY TABLES **17**

Supplementary Table 1 17

Supplementary Table 2 19

Supplementary Table 3 20

Supplementary Table 4 21

Supplementary Table 5 22

Supplementary Table 6 23

Supplementary Table 7 24

Supplementary Table 8 25

Supplementary Table 9 26

Supplementary Table 10 27

Supplementary Table 11 28

Supplementary Table 12 29

Supplementary Table 13 30

Supplementary Table 14 31

SUPPLEMENTARY NOTES **33**

VA Million Veteran Program COVID-19 Science Initiative: Core Acknowledgment for Publications 33

VA Million Veteran Program COVID-19 Science Initiative 33

Mount Sinai COVID-19 Biobank: Core Acknowledgment for Publications 37

Other Acknowledgements 38

DESCRIPTION OF THE SUPPLEMENTARY DATA FILES 38

SUPPLEMENTARY DATA 1 TO 7 38

SUPPLEMENTARY DATA 8 41

SUPPLEMENTARY DATA 9 42
SUPPLEMENTARY DATA 10 43
SUPPLEMENTARY RESULTS 43
COVID-19 phenotypes genetically regulated gene expression (GReX) comparison. 43
SUPPLEMENTARY REFERENCES 44

SUPPLEMENTARY FIGURES

Supplementary Fig. 1

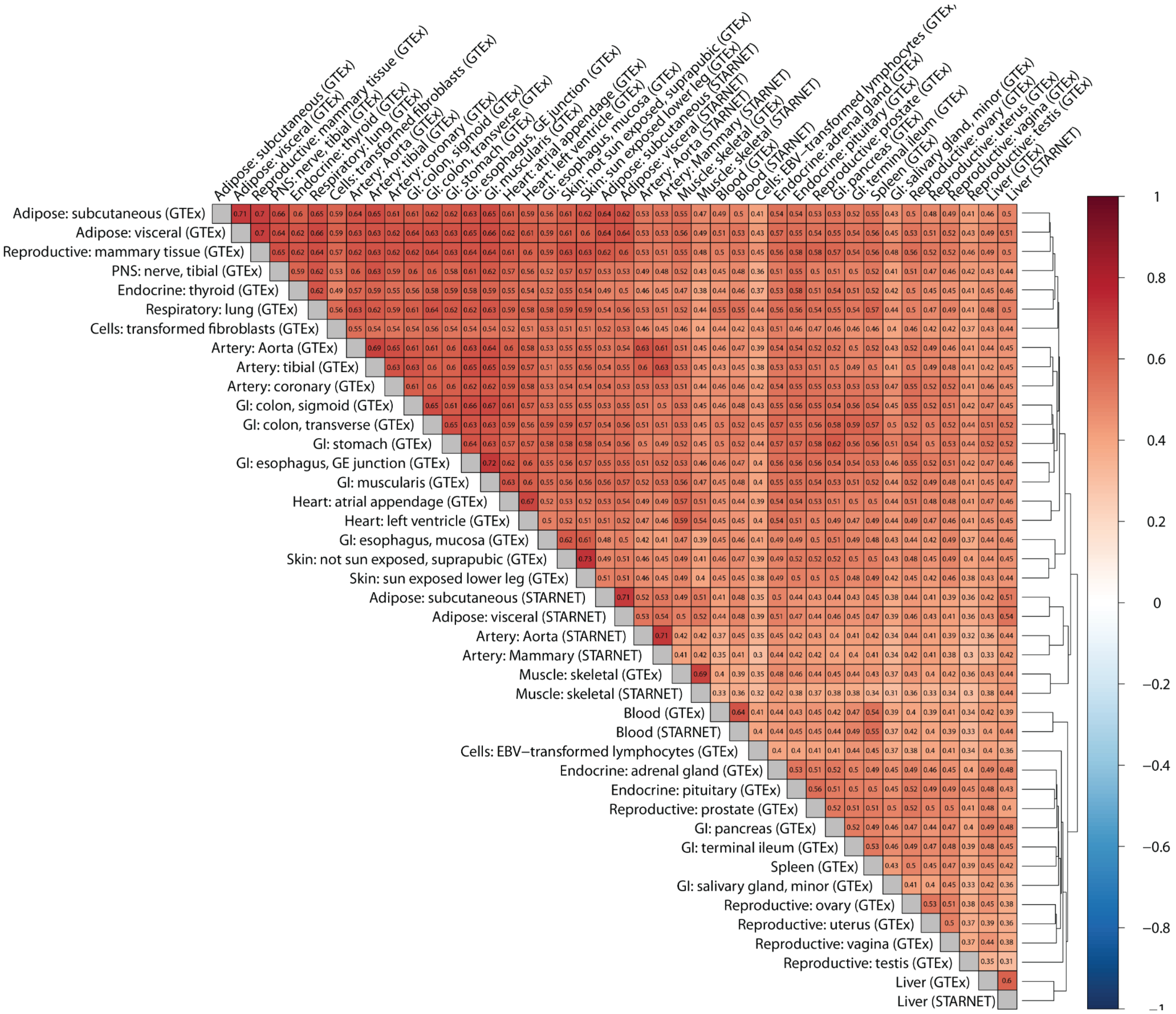

<u>Supplementary Fig. 1. Correlation of genetically regulated gene expression (GReX) across all tissues considering all COVID-19 phenotypes.</u> Correlation was calculated for imputed expression changes with the Pearson method. Dendrogram on the right edge is shown from Ward hierarchical clustering.

Supplementary Fig. 2

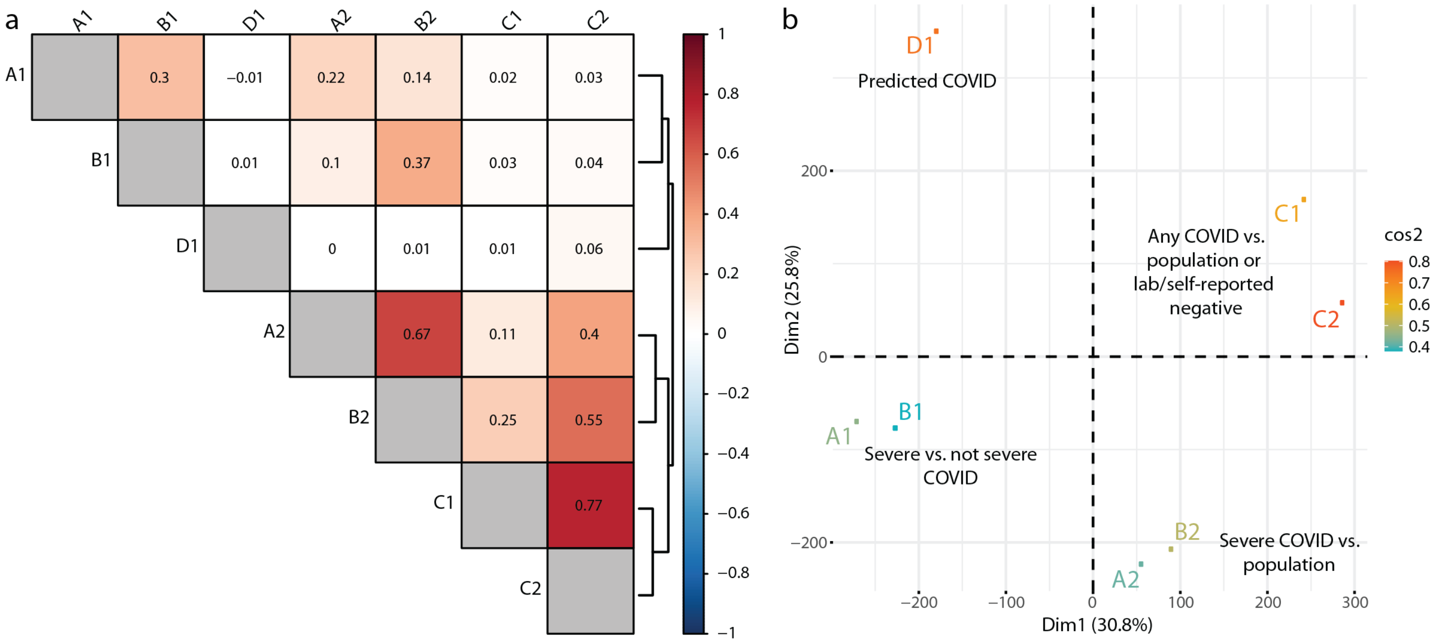

Supplementary Fig. 2. Comparison of COVID-19 GWAS phenotypes. Panel a. Correlation of GReX across COVID phenotypes taking into account all tissue models. Correlation was calculated for imputed expression changes with the Pearson method. Dendrogram on the right edge shows Ward hierarchical clustering. **Panel b.** PCA of GReX of COVID phenotypes showing clustering of phenotypes (e.g. A1&B1). The sums of the squared cosines of the first two principal components (PCs: Dim1 and Dim2) for each phenotype are color-coded as shown in the legend on the right and represent the importance of these PCs for each phenotype. A1: Very severe respiratory confirmed COVID vs. not hospitalized COVID ; A2: Very severe respiratory confirmed COVID vs. population; B1: Hospitalized COVID vs. not hospitalized COVID; B2: Hospitalized COVID vs. population; C1: COVID vs. lab/self-reported negative; C2: COVID vs. population; D1: predicted COVID from self-reported symptoms vs. predicted or self-reported non-COVID.

Supplementary Fig. 3

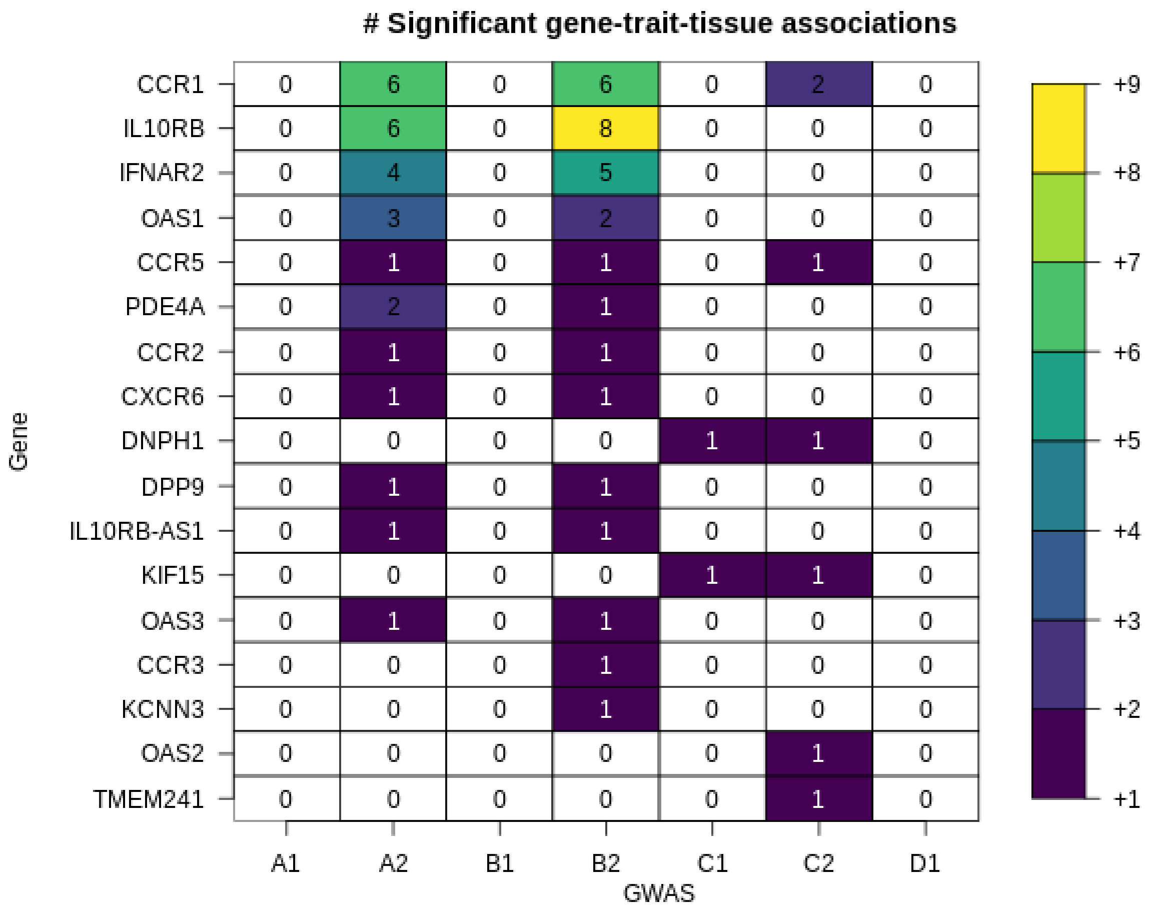

<u>Supplementary Fig. 3. TWAS gene-trait-tissue association counts per gene and COVID-19 phenotype (considering all tissue models).</u> Only FDR-significant associations are shown. A1: Very severe respiratory confirmed COVID vs. not hospitalized COVID; A2: Very severe respiratory confirdifferentmed COVID vs. population; B1: Hospitalized COVID vs. not hospitalized COVID; B2: Hospitalized COVID vs. population; C1: COVID vs. lab/self-reported negative; C2: COVID vs. population; D1: predicted COVID from self-reported symptoms vs. predicted or self-reported non-COVID.

Supplementary Fig. 4

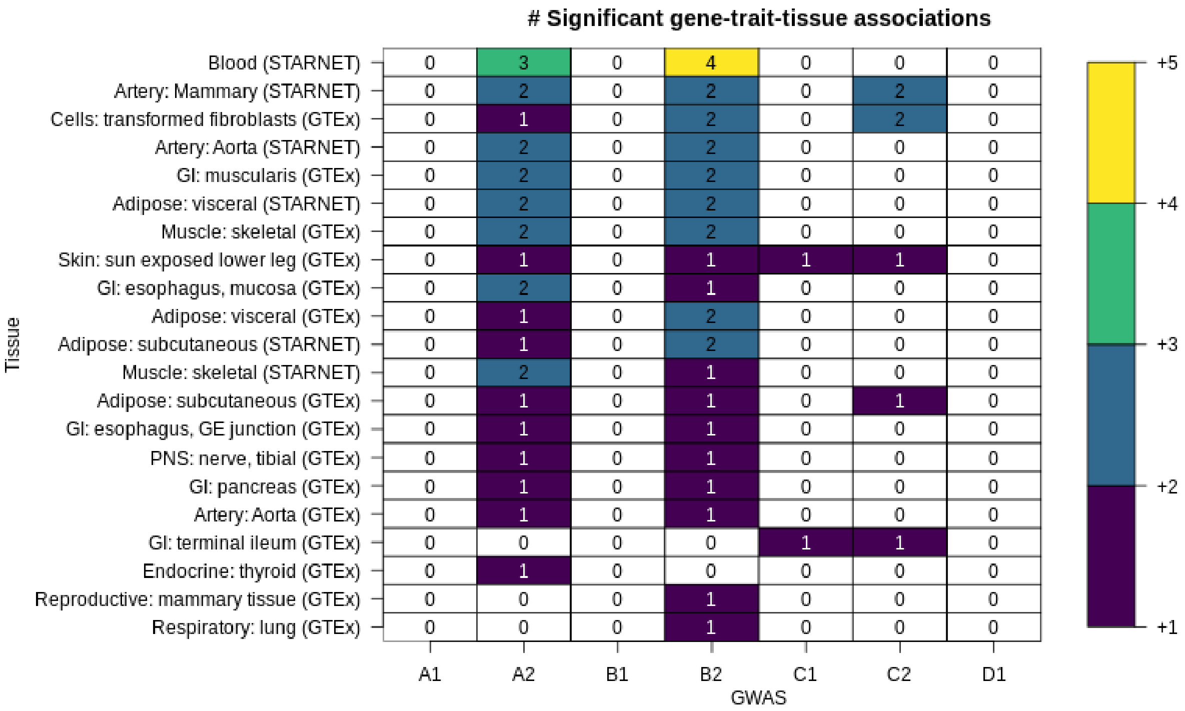

<u>Supplementary Fig. 4. TWAS gene-trait-tissue association counts per tissue and COVID-19 phenotype.</u> Only FDR-significant associations are shown. To estimate FDR-adjusted p values (significant if FDR-adjusted $p \leq 0.05$) we consider all phenotypes and tissues. A1: Very severe respiratory confirmed COVID vs. not hospitalized COVID ; A2: Very severe respiratory confirmed COVID vs. population; B1: Hospitalized COVID vs. not hospitalized COVID; B2: Hospitalized COVID vs. population; C1: COVID vs. lab/self-reported negative; C2: COVID vs. population; D1: predicted COVID from self-reported symptoms vs. predicted or self-reported non-COVID.

Supplementary Fig. 5

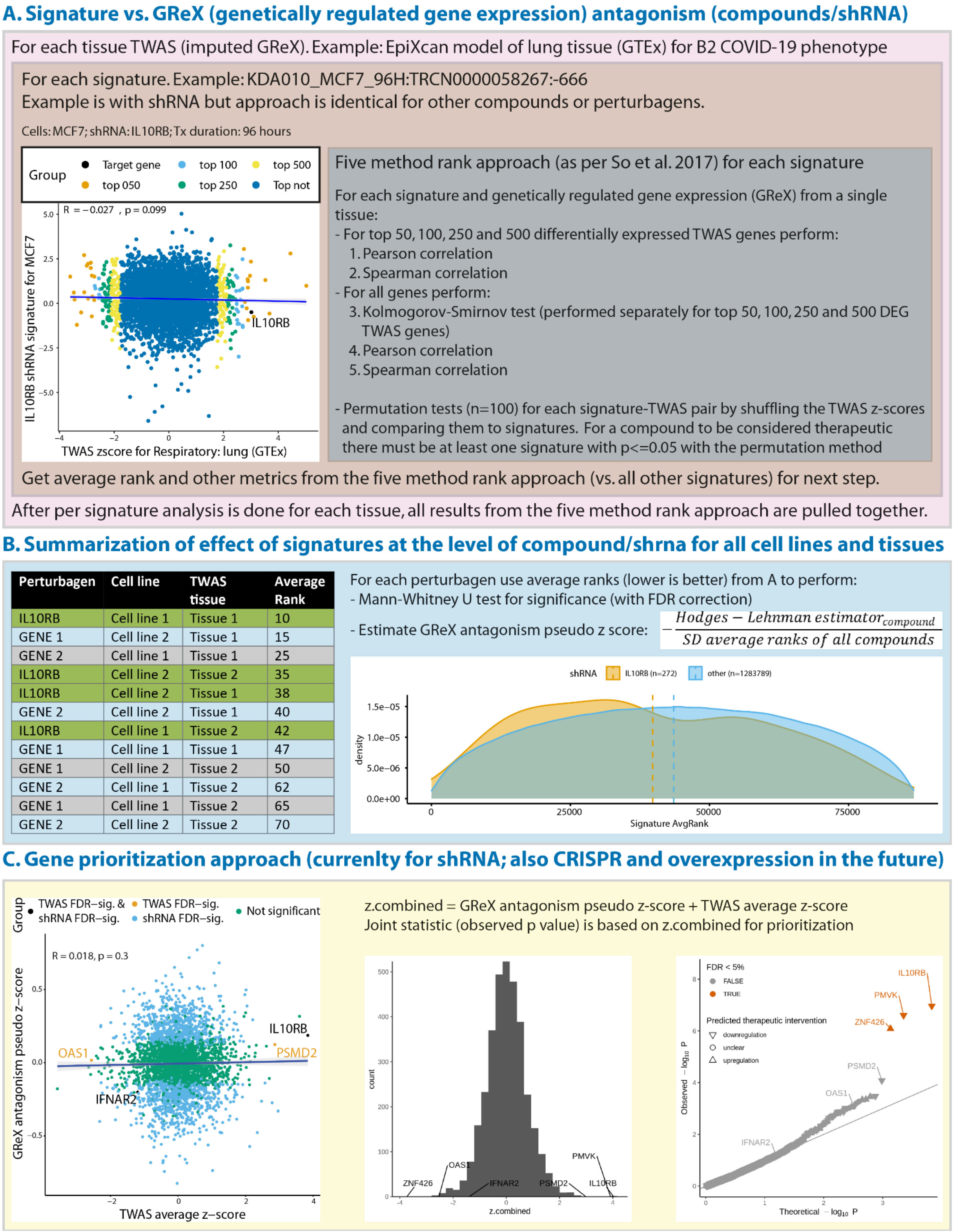

Supplementary Fig. 5. Gene target prioritization approach. **Panel A.** Each signature from the perturbagen signature library (e.g. *IL10RB* shRNA treatment for 96 hours in MCF7 cells)

was assessed for its ability to reverse the trait-associated imputed transcriptomes. **Panel B.** Signatures were grouped by peturbagen (shRNA) and we first tested whether the signatures for a specific perturbagen are more likely to be ranked higher or lower (Mann-Whitney U test); then we obtained a GReX antagonism pseudo z-score as follows: $-\frac{Hodges-Lehmann\ estimator_{perturbagen}}{SD\ average\ ranks\ of\ all\ perturbagens}$ (terms compound and peturbagen are used interchangeably). **Panel C.** Identification of candidate gene targets by integrating TWAS gene-trait associations and predicted effects of shRNAs in reversing COVID-19-associated transcriptomes. On the left (scatter plot), the x-axis corresponds to the average TWAS z-score (z_{TWAS}) across all EpiXcan tissues that had at least one FDR significant gene-trait association and the y-axis corresponds to the GReX antagonism pseudo z-score ($pseudo\ z_{GReX\ antagonism}$) which is defined as the negative Hodges-Lehmann estimator (of the median difference between that specific shRNA vs. all other shRNAs) divided by the standard deviation of the ranks of the compounds ($-\frac{Hodges-Lehmann\ estimator_{perturbagen}}{SD\ average\ ranks\ of\ all\ perturbagens}$). A positive pseudo z-score was interpreted as a potentially therapeutic shRNA whereas a negative pseudo z-score would suggest that the shRNA was not antagonizing the imputed transcriptome and is thus likely to exacerbate the phenotype. It is worth noting that we only generated shRNA gene expression signatures for 4,302 genes which is a subset of the genes that were reliably imputed from the TWAS. At the center, we see the histogram of the combined z scores ($z_{combined} = \overline{z_{TWAS}} + pseudo\ z_{GReX\ antagonism}$). On the right (QQ plot same as in Figure 2C), we show the p value corresponding to the joint statistic of the two approaches ($z_{combined}$) described above against the null. FDR-significant candidate genes are labelled orange (whereas non FDR-significant are grey) and we also provide the direction of the predicted therapeutic intervention when this can be determined (upregulation or downregulation). *IL10RB*, *PMVK* and *ZNF426* are the three FDR-significant target genes, *PSMD2*, *OAS1* and *IFNAR2* are also displayed since they were FDR-significant TWAS genes to demonstrate the added value of the approach.

Supplementary Fig. 6

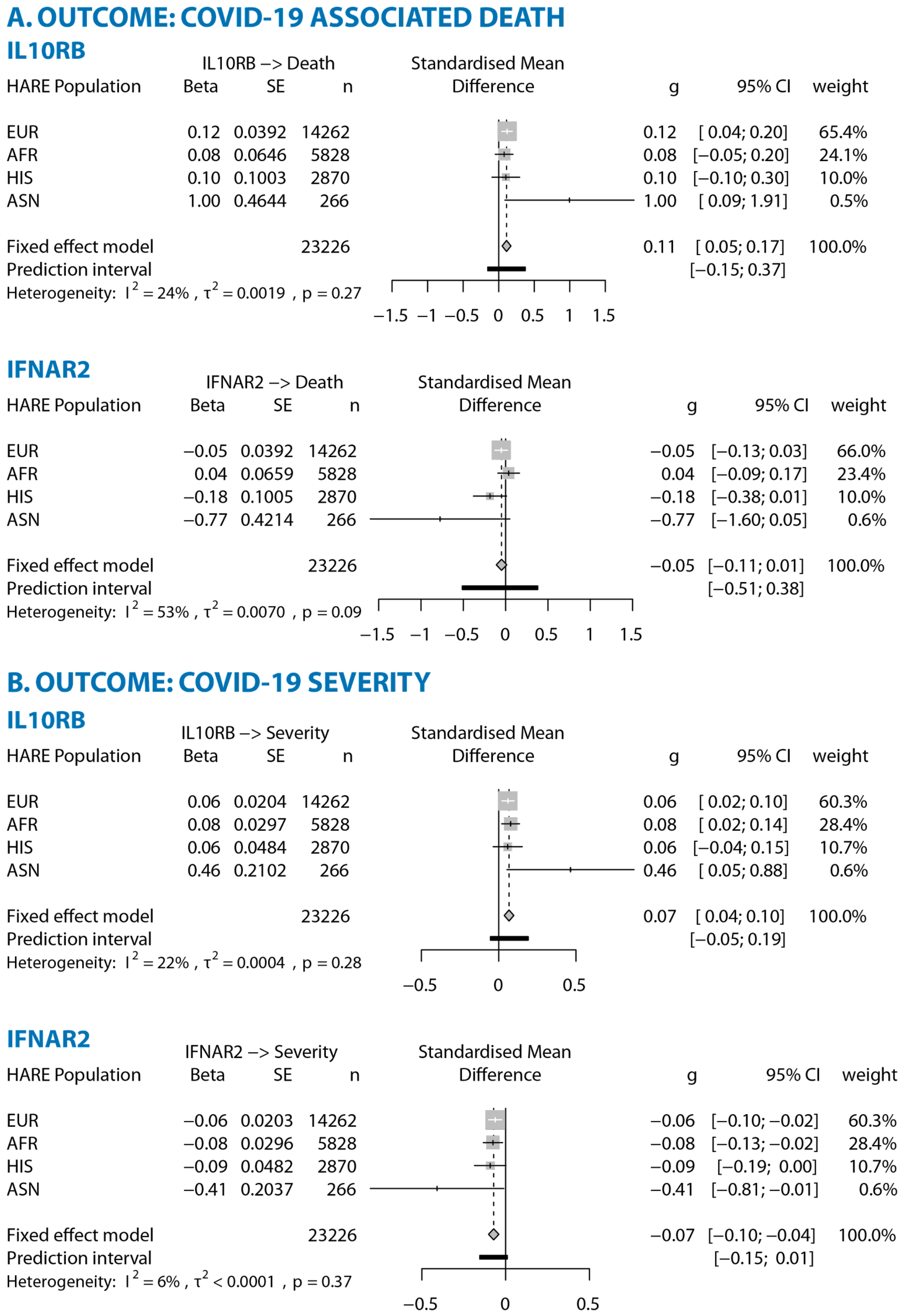

Supplementary Fig. 6. Transethnic meta-analysis for *IL10RB* and *IFNAR2* GReX with COVID-19 outcomes. Death (**A**) and severity score (**B**).

Supplementary Fig. 7

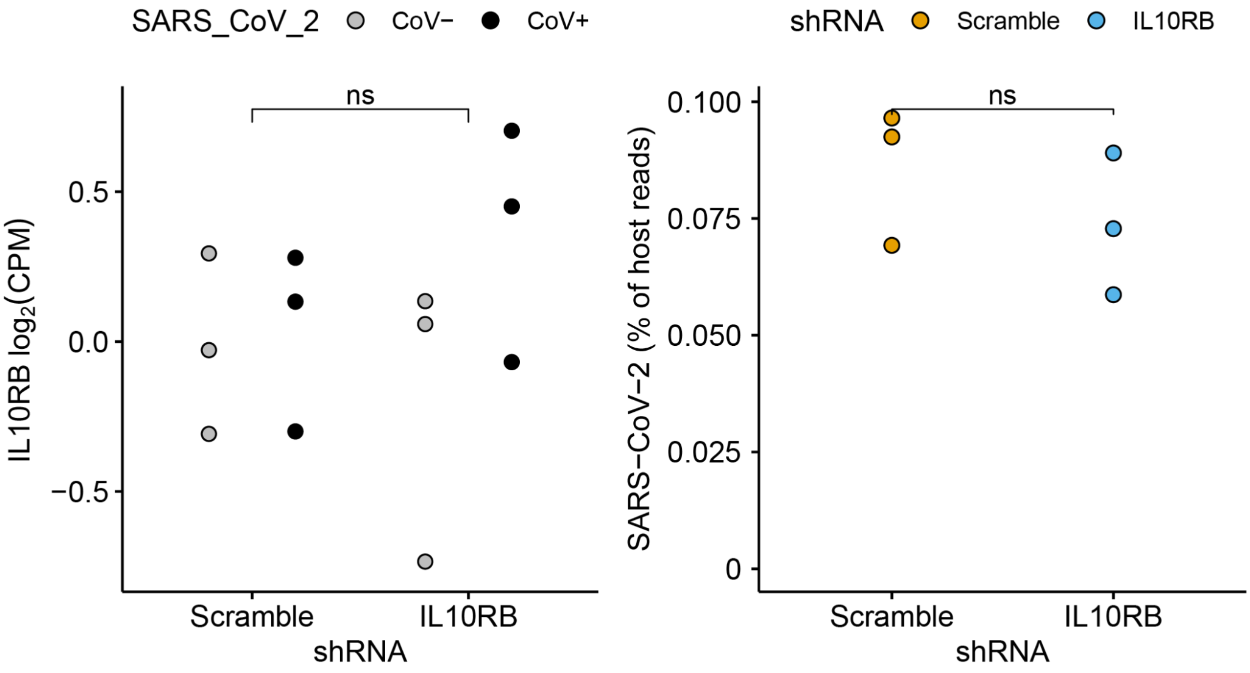

Supplementary Fig. 7. Effect of IL10RB shRNA on SARS-CoV-2 viral load in hiPSC-derived NGN-2 glutamatergic neurons. shRNA for IL10RB was used to knock-down *IL10RB* in hiPSC-derived NGN2-glutamatergic neurons. ***, ** and * correspond to p values from the linear model as ≤ 0.001, 0.01 and 0.05, respectively. For the SARS-CoV-2 viral load (right panel) we perform pairwise comparison with unpaired t-test; ***, ** and * correspond to p values of ≤ 0.001, 0.01 and 0.05, respectively. Even when considering only cells infected with SARS-CoV-2 (CoV+), there is no statistically significant difference in *IL10RB* expression (p = 0.3243, unpaired t-test).

Supplementary Fig. 8

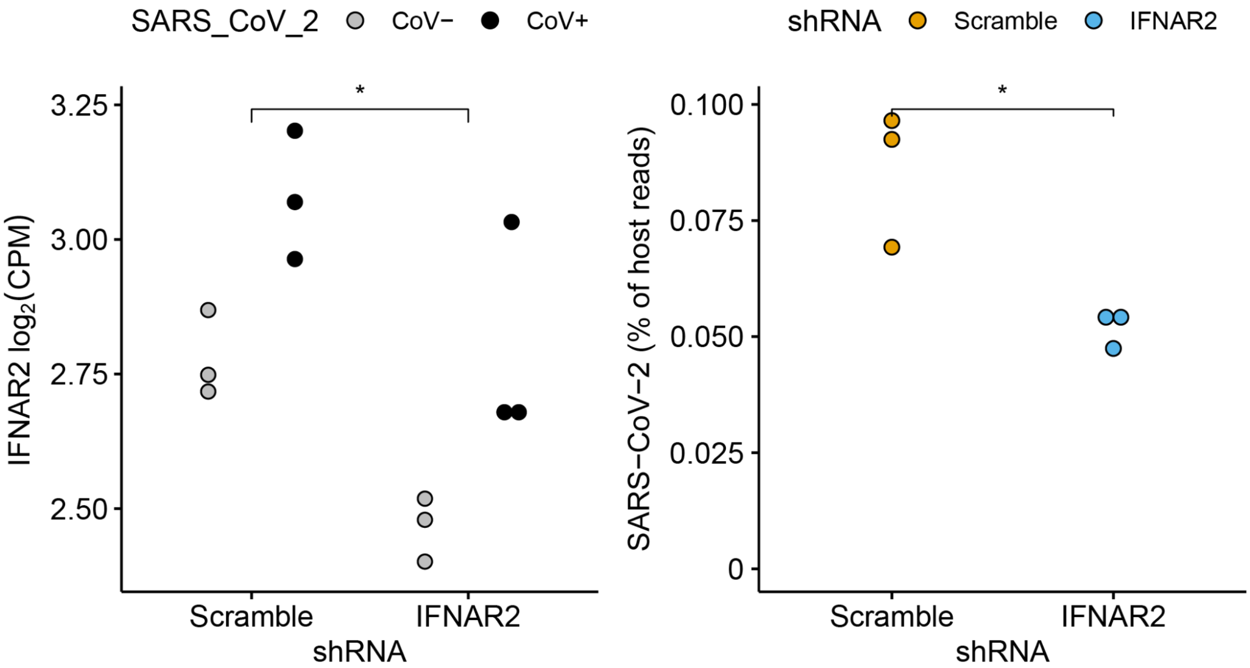

<u>Supplementary Fig. 8. Effect of IFNAR2 shRNA on SARS-CoV-2 viral load in hiPSC-derived NGN-2 glutamatergic neurons.</u> shRNAs for IFNAR2 were used to knock-down *IFNAR2* in hiPSC-derived NGN2-glutamatergic neurons. ***, ** and * correspond to p values from the linear model of ≤ 0.001, 0.01 and 0.05, respectively. For the SARS-CoV-2 viral load (right panel) we perform pairwise comparison with unpaired t-test; ***, ** and * correspond to p values of ≤ 0.001, 0.01 and 0.05, respectively.

Supplementary Fig. 9

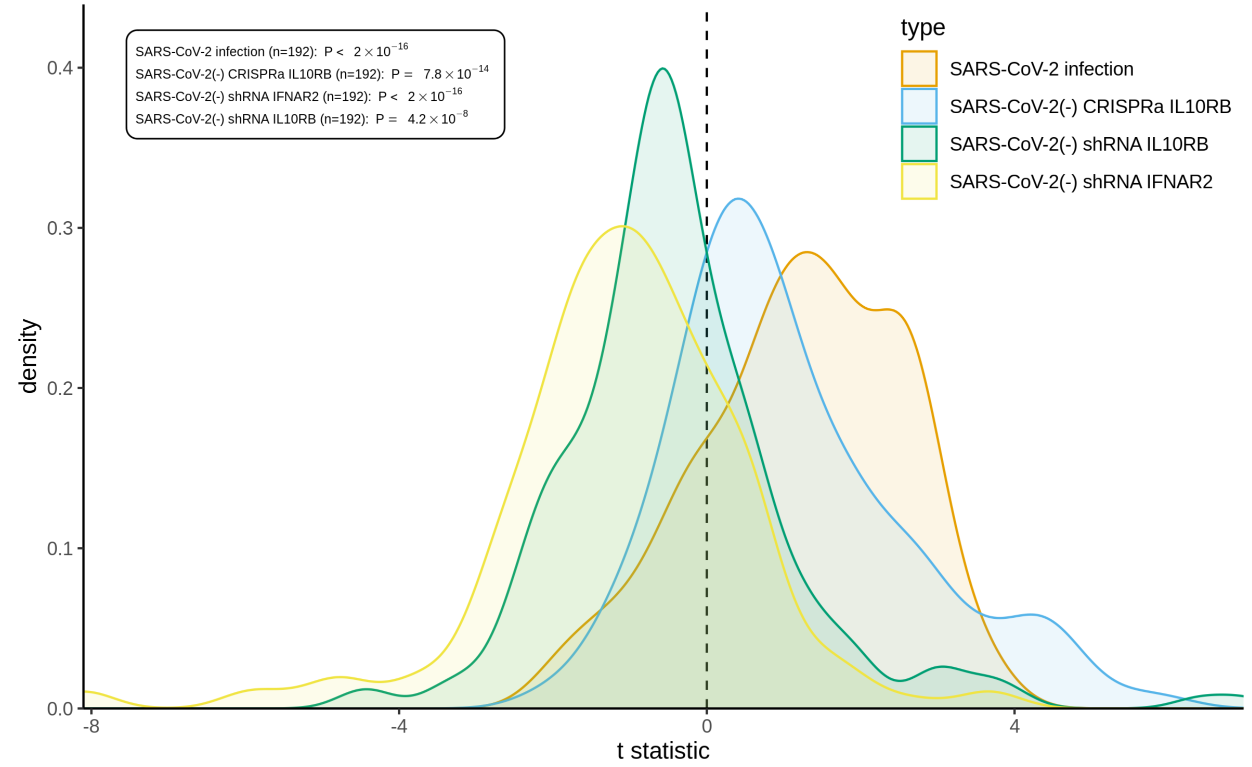

Supplementary Fig. 9. Competitive betacoronavirus gene set enrichment analysis in hiPSC-derived NGN-2 glutamatergic neurons. Distribution of competitive enrichment t statistics for gene sets that correspond to betacoronavirus relevant gene sets e.g. infections across different cell systems and tissues (n=192; pruned betacoronavirus gene sets with a Jaccard index filter of 0.2). P values are from sign test against a theoretical median of 0.

Supplementary Fig. 10

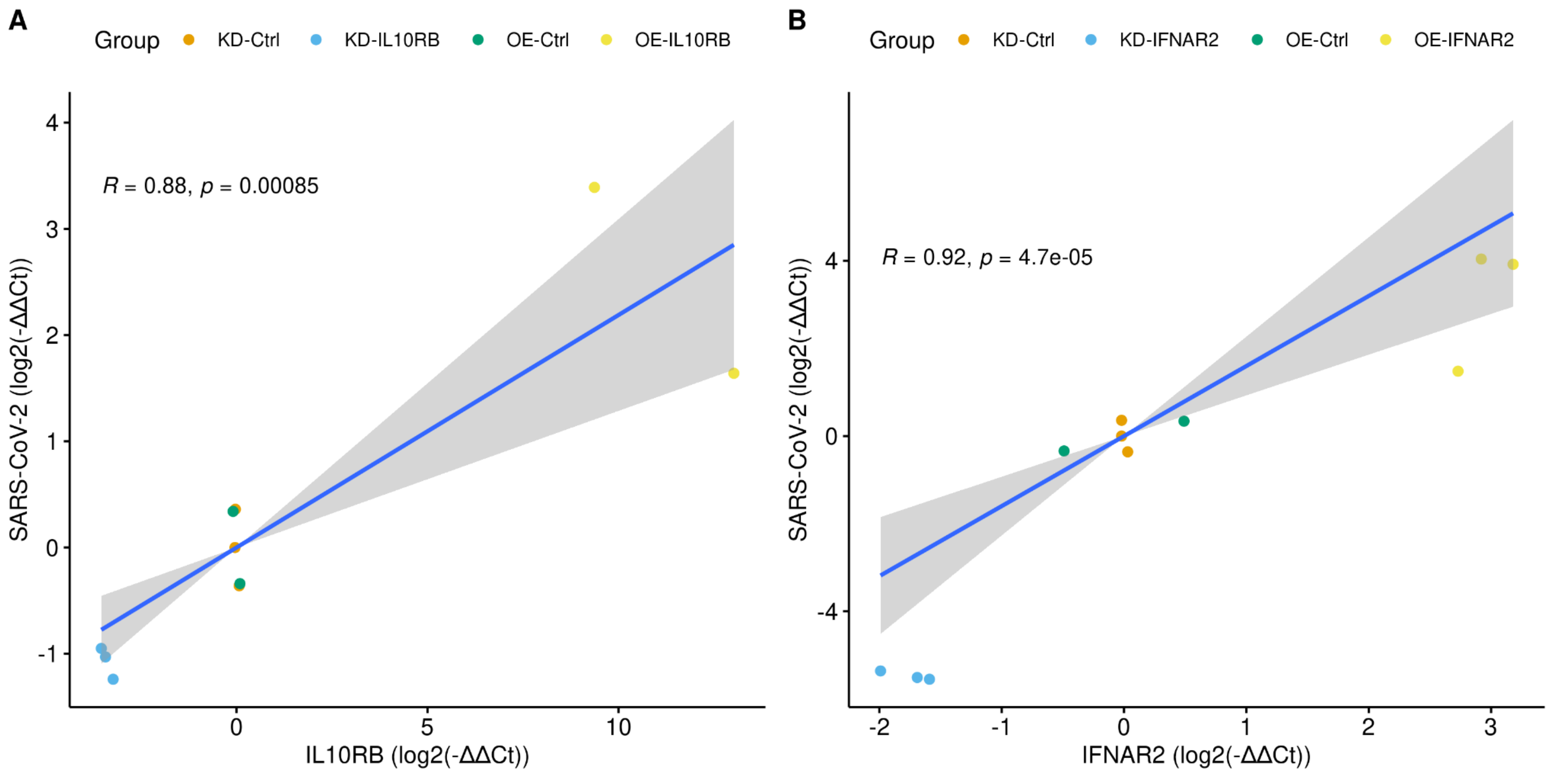

Supplementary Fig. 10. Effect of *IL10RB* and *IFNAR2* expression manipulation on SARS-CoV-2 viral load in A549-ACE2 alveolar cells. SARS-CoV-2 log2(-ΔΔCt) values reflecting the amount of SARS-CoV-2 S RNA in response to knock-down or overexpression of *IL10RB* (A) and *IFNAR2* (B). KD: knock-down from pooled siRNA transfection; OE: overexpression with pLVX.TetOne expression vector; statistical test: Pearson's correlation analysis. Quantification was performed using RT-qPCR and analyzed with the -ΔΔCt method and values were normalized against their respective controls (Ctrl): non-infected cells for *IL10RB* and *IFNAR2* expression levels (x-axes) and SARS-CoV-2 infected cells for SARS-CoV-2 viral load (y-axes). Within the siRNA subgroup, controls were cells transfected with non-targeting control siRNA and infected with SARS-CoV-2 at an MOI of 0.02 for 48 hours. Within the overexpression subgroup, controls were cells treated with doxycycline to induce expression of 2x-strept-eGFP, and infected with SARS-CoV-2 at an MOI of 0.02 for 48 hours. For pairwise comparisons, see Supplementary Table 10.

Supplementary Fig. 11

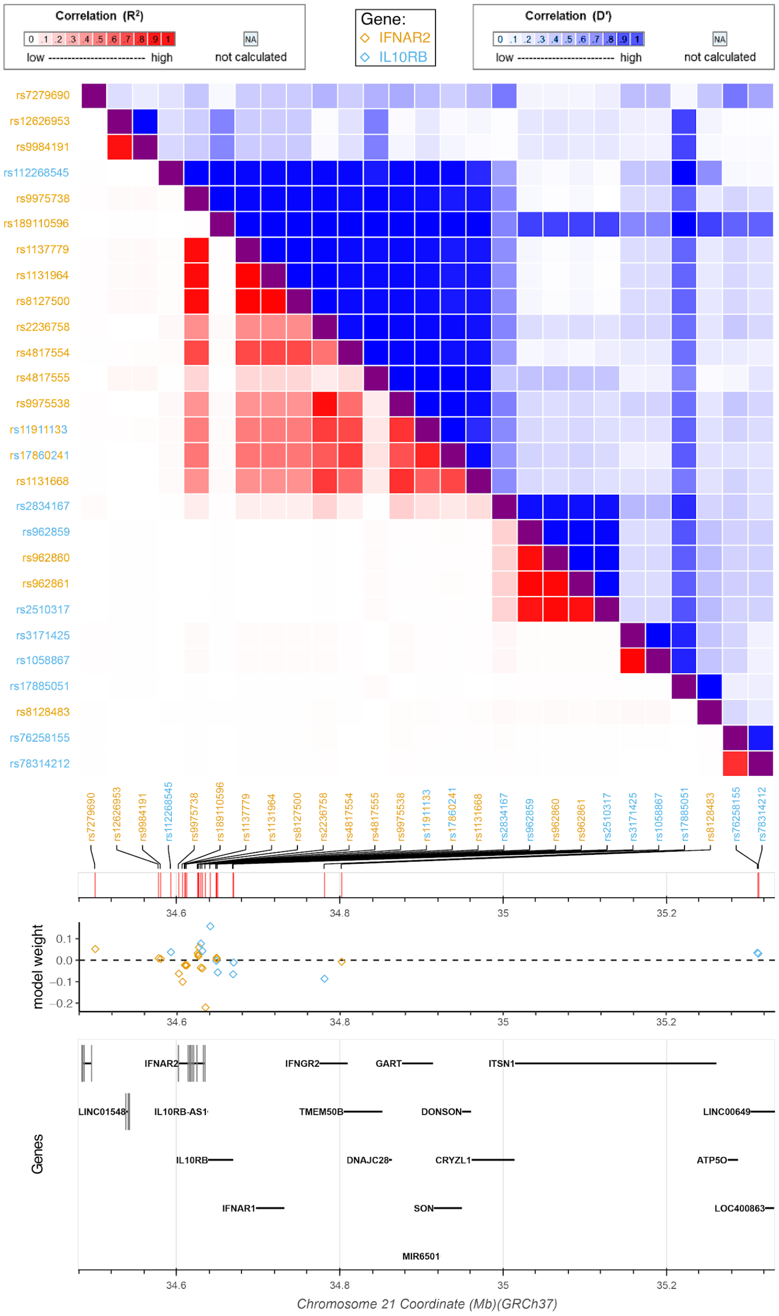

Supplementary Fig. 11. Correlation map of SNPs with sizable contribution to the Blood (STARNET) models of *IFNAR2* and *IL10RB*. SNPs that were used for *IFNAR2* are orange, *IL10RB* are blue and those used by both have these two colors alternating by letter. The top panel shows SNP correlation (R^2 and D'), the middle panel shows the model weights and the bottom panel the genes in the region. Only SNPs with a model prior ≥ 2 for each model are shown; SNP correlation based on 1000G reference panel.

Supplementary Fig. 12

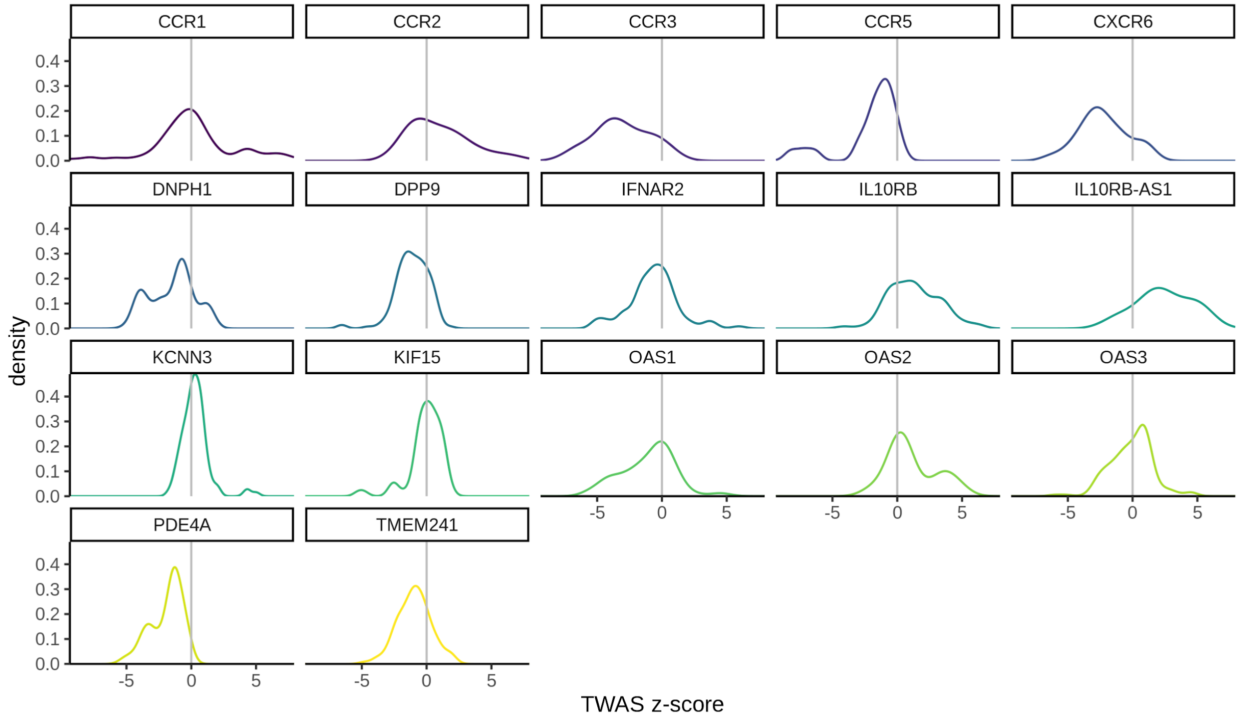

Supplementary Fig. 12. Density plots of TWAS association z-scores for FDR-significant genes (across all 7 COVID-19 phenotypes and 42 tissues). For some genes, such as *IL10RB,* there was a relatively consistent shift of the z-scores to one direction (e.g. right) whereas other genes, such as *IFNAR2,* showed both low and high z-score values suggesting phenotype and/or tissue specificity. FDR-significant genes (FDR-adjusted p≤0.05) for all COVID-19 phenotypes and tissues are displayed.

Supplementary Fig. 13

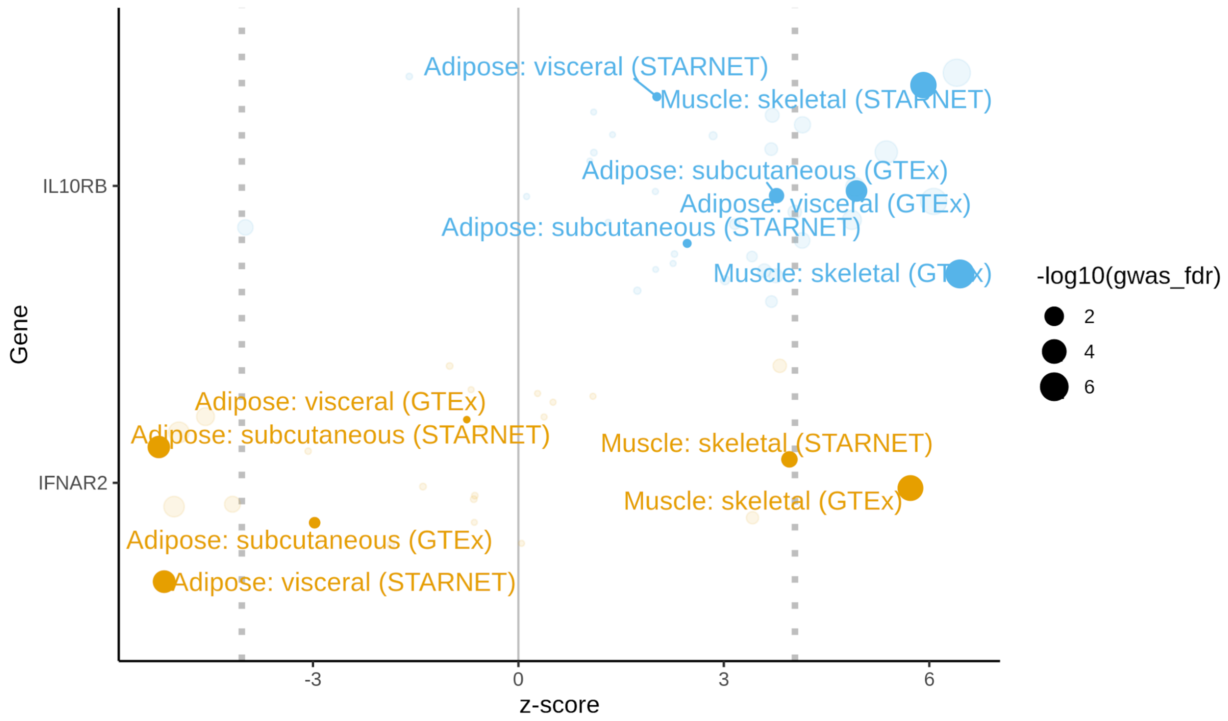

Supplementary Fig. 13. Comparing tissue specificity for adipose and muscle tissues of *IL10RB* and *IFNAR2* TWAS z-scores. *IFNAR2* (orange) TWAS z-scores are consistently low for adipose tissue and high for skeletal muscle - this effect is consistent for related tissues (e.g. visceral and subcutaneous adipose tissue) and across cohorts (STARNET and GTEx). No such tissue specificity is observed in *IL10RB* (blue). Only the B2 phenotype for COVID-19 associated hospitalization was considered (FDR for B2 is displayed on the right) and FDR significance levels for z-scores are denoted with vertical dotted lines. Tissue z-scores not corresponding to adipose or skeletal muscle tissue are faded. It is worth noting that the discordant faded blue dot (*IL10RB*) with a negative z-score close to significance (<-3) doesn't correspond to an endogenous tissue (transformed fibroblasts, GTEx).

SUPPLEMENTARY TABLES

Supplementary Table 1

Category: tissue/cell (Cohort)	Transcriptomic imputation method used	Used in gene targeting and drug repurposing analysis?
Adipose: subcutaneous (GTEx)	EpiXcan	Yes
Adipose: subcutaneous (STARNET)	EpiXcan	Yes
Adipose: visceral (GTEx)	EpiXcan	Yes
Adipose: visceral (STARNET)	EpiXcan	Yes
Artery: Aorta (GTEx)	EpiXcan	Yes
Artery: Aorta (STARNET)	EpiXcan	Yes
Artery: coronary (GTEx)	EpiXcan	No
Artery: Mammary (STARNET)	EpiXcan	Yes
Artery: tibial (GTEx)	PrediXcan	No
Blood (GTEx)	EpiXcan	No
Blood (STARNET)	EpiXcan	Yes
Cells: EBV-transformed lymphocytes (GTEx)	PrediXcan	No
Cells: transformed fibroblasts (GTEx)	PrediXcan	No
Endocrine: adrenal gland (GTEx)	EpiXcan	No
Endocrine: pituitary (GTEx)	PrediXcan	No
Endocrine: thyroid (GTEx)	PrediXcan	No
GI: colon, sigmoid (GTEx)	EpiXcan	No
GI: colon, transverse (GTEx)	EpiXcan	No
GI: esophagus, GE junction (GTEx)	EpiXcan	Yes
GI: esophagus, mucosa (GTEx)	EpiXcan	Yes
GI: muscularis (GTEx)	EpiXcan	Yes
GI: pancreas (GTEx)	EpiXcan	Yes
GI: salivary gland, minor (GTEx)	PrediXcan	No
GI: stomach (GTEx)	EpiXcan	No
GI: terminal ileum (GTEx)	EpiXcan	No
Heart: atrial appendage (GTEx)	EpiXcan	No
Heart: left ventricle (GTEx)	EpiXcan	No
Liver (GTEx)	EpiXcan	No
Liver (STARNET)	EpiXcan	No
Muscle: skeletal (GTEx)	EpiXcan	Yes
Muscle: skeletal (STARNET)	EpiXcan	Yes
PNS: nerve, tibial (GTEx)	PrediXcan	No
Reproductive: mammary tissue (GTEx)	EpiXcan	Yes
Reproductive: ovary (GTEx)	EpiXcan	No
Reproductive: prostate (GTEx)	PrediXcan	No
Reproductive: testis (GTEx)	PrediXcan	No
Reproductive: uterus (GTEx)	PrediXcan	No
Reproductive: vagina (GTEx)	PrediXcan	No
Respiratory: lung (GTEx)	EpiXcan	Yes
Skin: not sun exposed, suprapubic (GTEx)	EpiXcan	No

Skin: sun exposed lower leg (GTEx)	EpiXcan	Yes
Spleen (GTEx)	EpiXcan	No

Supplementary Table 1. The 42 transcriptomic imputation models used in this study. Information is also provided regarding which imputation method was used and whether it was used for the gene targeting and drug repurposing pipelines.

Supplementary Table 2

Short name	Phenotype	n_{cases}	$n_{controls}$	Ancestry superpopulation background	GWAS	TWAS results in
A1	Very severe respiratory confirmed covid vs. not hospitalized covid	269	688	EUR	“A1_ALL”	Supplementary Data 1
A2	Very severe respiratory confirmed COVID vs. population	4,336	623,902	EUR + AMR	“A2_ALL_leave_23andme”	Supplementary Data 2
B1	Hospitalized COVID vs. not hospitalized COVID	2,430	8,478	ALL except EAS	“B1_ALL”	Supplementary Data 3
B2	Hospitalized COVID vs. population	6,406	902,088	EUR	“B2_ALL_eur_leave_23andme”	Supplementary Data 4
C1	COVID vs. lab/self-reported negative	8,668	101,861	ALL except EAS	“C1_ALL_leave_23andme”	Supplementary Data 5
C2	COVID vs. population	14,134	1,284,876	EUR	“C2_ALL_eur_leave_23andme”	Supplementary Data 6
D1	predicted COVID from self-reported symptoms vs. predicted or self-reported non-COVID	3,204	35,728	EUR	“D1_ALL”	Supplementary Data 7

Supplementary Table 2. Overview of the GWAS summary statistics that were used. Column “Short name”: short name of the phenotype; column “Phenotype”: description of the phenotype; Columns “n_{cases}” and “$n_{controls}$” correspond to the number of cases and controls used in this study; column “Ancestry superpopulation background”: ancestry superpopulations that were included in the GWAS; column “GWAS”: GWAS summary statistics used; column “TWAS results in”: Supplementary Data file where the TWAS results can be found. “EUR”, “AMR”, “EAS” stand for European, admixed American and East Asian ancestries. “ALL” refers to all the superpopulations, as defined by the 1000 genomes project which includes the above plus African and South Asian ancestries.

Supplementary Table 3

	Severity Analysis Cohort					PheWAS Cohort
	EUR	AFR	HIS	ASN	ALL	EUR
Sample Size (%)	14,262 (61.4%)	5,828 (25.1%)	2,870 (12.4%)	266 (1.1%)	23,226 (100%)	296,407 (N/A)
Median Age (IQR)	71 (16)	64 (17)	60 (27)	50 (27)	68 (18)	71 (14)
Female (%)	1,231 (8.6%)	824 (14.1%)	295 (10.3%)	26 (9.8%)	2,376 (10.2%)	21,084 (7.1%)
Median Elixhauser (2yr) (IQR)	4 (13)	5 (14)	0 (8)	0 (4)	4 (13)	N/A
COVID Severity<br>- Mild (%)<br>- Median (%)<br>- Severe (%)<br>- Death (%)	 10,851 (76.1%)<br>2,301 (16.1%)<br>383 (2.7%)<br>727 (5.1%)	 4,113 (70.6%)<br>1,187 (20.4%)<br>266 (4.6%)<br>262 (4.5%)	 2,221 (77.4%)<br>439 (15.3%)<br>93 (3.2%)<br>117 (4.1%)	 217 (81.6%)<br>26 (9.8%)<br>8 (3.0%)<br>15 (5.6%)	 17,402 (74.9%)<br>3,953 (17.0%)<br>750 (3.2%)<br>1,121 (4.8%)	N/A
Median ICD Count (IQR)	N/A	N/A	N/A	N/A	N/A	136 (175)
Median length of record (IQR)	N/A	N/A	N/A	N/A	N/A	4,575.4 (3,350.0)

Supplementary Table 3. Demographic characteristics of the MVP cohorts used in the GReX association with COVID-19 severity and death, and PheWAS.

Supplementary Table 4

Gene	Population	n	Beta	SE	P	Bonferroni-adjusted p
IL10RB	ALL	23226	0.067	0.016	**2.4×10^{-05}**	**9.8×10^{-05}**
	EUR	14262	0.060	0.020	**3.3×10^{-03}**	**1.3×10^{-02}**
	AFR	5828	0.077	0.030	**9.6×10^{-03}**	**3.8×10^{-02}**
	HIS	2870	0.058	0.048	2.3×10^{-01}	9.2×10^{-01}
	ASN	266	0.465	0.210	**2.7×10^{-02}**	1.1×10^{-01}
IFNAR2	ALL	23226	-0.071	0.016	**6.2×10^{-06}**	**2.5×10^{-05}**
	EUR	14262	-0.062	0.020	**2.3×10^{-03}**	**9.0×10^{-03}**
	AFR	5828	-0.076	0.030	**1.0×10^{-02}**	**4.1×10^{-02}**
	HIS	2870	-0.092	0.048	5.5×10^{-02}	2.2×10^{-01}
	ASN	266	-0.409	0.204	**4.4×10^{-02}**	1.8×10^{-01}

Supplementary Table 4. GReX association with COVID-19 severity. Bonferroni-adjustment is performed for $n_{genes} \times n_{outcomes} = 4$ for each population cohort.

Supplementary Table 5

Population / ethnicity	Sample Size (%)	Median Age (IQR)	Female (%)
White / Hispanic or Latino	52 (9.2%)	66 (21.25)	24 (46.2%)
Black or African American / Hispanic or Latino	10 (1.8%)	52 (32)	7 (70%)
Unknown / Hispanic or Latino	107 (18.8%)	64 (25)	41 (38.3%)
More Than One Race / Hispanic or Latino	15 (2.6%)	64 (18)	7 (46.7%)
American Indian or Alaska Native / Hispanic or Latino	4 (0.7%)	63.5 (17.5)	1 (25%)
White / not Hispanic or Latino	128 (22.5%)	68 (28.5)	54 (42.2%)
Black or African American / not Hispanic or Latino	106 (18.7%)	63 (16.5)	54 (50.9%)
Unknown / not Hispanic or Latino	12 (2.1%)	62 (14.5)	1 (8.3%)
More Than One Race / not Hispanic or Latino	9 (1.6%)	56 (12)	4 (44.4%)
Asian / not Hispanic or Latino	36 (6.3%)	60 (22.25)	11 (30.6%)
American Indian or Alaska Native / not Hispanic or Latino	3 (0.5%)	62 (7.5)	0 (0%)
Native Hawaiian or Other Pacific Islander / not Hispanic or Latino	1 (0.2%)	68 (0)	1 (100%)
White / unknown	3 (0.5%)	88 (4.5)	2 (66.7%)
Unknown unknown	82 (14.4%)	62 (21.5)	35 (42.7%)

Supplementary Table 5. Demographic characteristics of the Mount Sinai COVID-19 Biobank.

Supplementary Table 6

	Count (%)
# Individuals with number of samples below:	
- 1	176 (31.0%)
- 2	156 (27.5%)
- 3	110 (19.4%)
- 4	71 (12.5%)
- 5	39 (6.9%)
- 6	15 (2.6%)
- 7	1 (0.2%)
# of samples with COVID Severity below:	
- Control	122 (10.1%)
- Moderate COVID-19	600 (49.6%)
- Severe COVID-19	269 (22.2%)
- Severe COVID-19 with EOD	218 (18.0%)

Supplementary Table 6. Sample characteristics of the Mount Sinai COVID-19 Biobank. The differential gene expression analysis was based on samples not individuals. Here we provide information about how many samples were taken from each individual and a breakdown by severity for the samples.

Supplementary Table 7

gRNA	Target gene	logFC	t	P value
IL10RB-1	*IL10RB*	2.67	5.67	0.0000009
IL10RB-2	*IL10RB*	1.46	3.44	0.0012
IL10RB-3	*IL10RB*	1.67	3.90	0.0003
IL10RB-4	*IL10RB*	0.98	3.64	0.0007

Supplementary Table 7. Effect of CRISPRa gRNAs on target gene. Metrics for scrambled gRNA.

Supplementary Table 8

shRNA	Target gene	logFC	t	P value
IL10RB	*IL10RB*	0.06	0.27	0.78
IFNAR2	*IFNAR2*	-0.41	-2.39	0.02

Supplementary Table 8. Effect of shRNAs on target genes. Metrics for scrambled shRNA

Supplementary Table 9

Treatment	Target gene	logFC	t	P value
SARS-CoV-2 infection	*IL10RB*	0.025	0.13	0.89
SARS-CoV-2 infection	*IFNAR2*	0.3	3.70	0.00098

Supplementary Table 9. Effect of SARS-CoV-2 infection on target genes (*IL10RB* and *IFNAR2*). Metrics for non SARS-CoV-2 infected cells taking into account (scrambled gRNA and shRNA treatments).

Supplementary Table 10

Treatment group	Target gene	SARS-CoV-2 infection	qPCR target	% control (-ΔΔCt method)	P value	Comparison
siRNA	*IL10RB*	No	*IL10RB*	9.5%	**4.0×10^{-06}**	a-IL10RB (n=3) vs. control siRNA (n=3)
		Yes	SARS-CoV-2	47.7%	**0.0088**	a-IL10RB (n=3) vs. control siRNA (n=3)
	IFNAR2	No	*IFNAR2*	29.9%	**0.00098**	a-IFNARB (n=3) vs. control siRNA (n=3)
		Yes	SARS-CoV-2	2.3%	**1.4×10^{-05}**	a-IFNARB (n=3) vs. control siRNA (n=3)
Exogenous expression	*IL10RB*	No	*IL10RB*	308,504.5%	**0.013**	IL10RB (n=3) vs. GFP induction (n=2); Dox+
		Yes	SARS-CoV-2	681.6%	0.11	IL10RB (n=2) vs. GFP induction (n=2); Dox+
	IFNAR2	No	*IFNAR2*	775.5%	**0.0053**	IFNAR2 (n=3) vs. GFP induction (n=2); Dox+
		Yes	SARS-CoV-2	1143.7%	0.06	IFNAR2 (n=3) vs. GFP induction (n=2); Dox+

Supplementary Table 10. *In vitro* manipulation of IL10RB and IFNAR2 expression in A549 alveolar cells exogenously expressing ACE2 (A549-ACE2). Treatment group: either siRNA or overexpression (doxycycline induction of stable cell lines) experiments; Target gene: gene target of knock-down or overexpression; SARS-CoV-2 infection: whether cells were infected with SARS-CoV-2 or not; qPCR target: qPCR amplification target - measurements for which statistical tests were performed; details for statistical tests are provided in the remaining columns. We performed pairwise comparisons with unpaired t-test.

Supplementary Table 11

Gene ($n_{\text{SNPs in model}}$)	$n_{\text{SNPs in EUR}}$	$n_{\text{SNPs in AFR}}$	$n_{\text{SNPs in HIS}}$	$n_{\text{SNPs in ASN}}$
IL10RB (20)	15	15	17	11
IFNAR2 (36)	20	15	20	16

Supplementary Table 11. Comparison of the number of SNP predictors present across different ancestral groups from the blood transcriptomic imputation model (STARNET) in MVP.

Supplementary Table 12

Severity	Description	n_{EUR}	n_{AFR}	n_{HIS}	n_{ASN}
Mild	SARS-CoV-2+	10,851	4,113	2,221	217
Moderate	SARS-CoV-2+ and hospitalized with or without low flow oxygen therapy	2,301	1,187	439	26
Severe	SARS-CoV-2+ and hospitalized with either ventilation, intubation, extracorporeal membrane oxygenation (EMCO), dialysis vasopressors or high flow oxygen therapy	383	266	93	8
Death	COVID-19 related death	727	262	117	15

Supplementary Table 12. COVID severity scale developed by VINCI and the MVP COVID-19 Science Initiative. Description and counts for each HARE-based population.

Supplementary Table 13

Severity	Description
Control	No COVID-19
Moderate	COVID-19 with abnormal (<94%) O2 saturation or pneumonia on imaging
Severe	COVID-19 with use of high-flow nasal cannula (HFNC), non-rebreather mask (NRB), bilevel positive airway pressure (BIPAP) or mechanical ventilation and no vasopressor use, and based on CrCl greater than 30 and alanine aminotransferase (ALT) less than 5× the upper limit of normal.
Severe with end-organ damage	COVID-19 as Severe but with use of vasopressors, or based on CrCl less than 30, new renal replacement therapy (hemodialysis/continuous veno-venous hemofiltration) or ALT more than 5× the upper limit of normal

Supplementary Table 13. COVID severity scale developed by the Mount Sinai COVID-19 Biobank. Severity score has been previously characterized in detail[1].

Supplementary Table 14

Experiment	Sequence name	Oligo Sequences
**NGN2 - *IL10RB* CRISPRa**	***IL10RB* gRNA#1**	caccgAGGCTTGGCAGATGCACACG / aaacCGTGTGCATCTGCCAAGCCTc (forward / reverse)
	***IL10RB* gRNA#2**	caccgGGATCCTCGCAAGCTTTGAA / aaacTTCAAAGCTTGCGAGGATCCc (forward / reverse)
	***IL10RB* gRNA#3**	caccgGCATGCTGGAATGACGGTGG / aaacCCACCGTCATTCCAGCATGCc (forward / reverse)
	***IL10RB* gRNA#4**	caccgTTGAAGTCCGCTCTCCGCAC / aaacGTGCGGAGAGCGGACTTCAAc (forward / reverse)
	**Scramble gRNA**	caccgGCACTCACATCGCTACATCA / aaacTGATGTAGCGATGTGAGTGCC (forward / reverse)
**NGN2 - *IL10RB* shRNA**	**SHCLNG-NM_000628 (Sigma)**	CCTGTGGATGACACCATTATT
**NGN2 - *IFNAR2* shRNA**	**SHCLNG-NM_000874 (Sigma)**	GCAGTAATAAAGTCTCCCTTA
**A549 - *IL10RB* siRNA: M-007926-02-0005 siGENOME Human IL10RB (3588) siRNA - SMARTpool**	**D-007926-03**	GCAAACAACCCAUGACGAA
	**D-007926-04**	GACCACACCUUGAGAGUCA
	**D-007926-05**	CAGCUCAGUACCUAAGUUA
	**D-007926-18**	CUACACAGAGCACGGACUU
**A549 - *IL10RB* siRNA: M-015411-00-0005 siGENOME Human IFNAR2 (3455) siRNA - SMARTpool**	**D-015411-01**	GGUGAAAUUUCCAUCUAUU
	**D-015411-02**	CAGAGGGAAUUGUUAAGAA
	**D-015411-03**	GAGCAAGCAGUAAUAAAGU
	**D-015411-04**	GAAGAUUUGAAGGUGGUUA

**A549 - *IL10RB* overexpression**	**gBlocks gene fragment for *IL10RB***	ATGGCGTGGAGCCTTGGGAGCTGGCTGGGTGGCTGCCTGCTGGTGTCAGCATTGGGAATGGT<br>ACCACCTCCCGAAAATGTCAGAATGAATTCTGTTAATTTCAAGAACATTCTACAGTGGGAGTCAC<br>CTGCTTTTGCCAAAGGGAACCTGACTTTCACAGCTCAGTACCTAAGTTATAGGATATTCCAAGAT<br>AAATGCATGAATACTACCTTGACGGAATGTGATTTCTCAAGTCTTTCCAAGTATGGTGACCACAC<br>CTTGAGAGTCAGGGCTGAATTTGCAGATGAGCATTCAGACTGGGTAAACATCACCTTCTGTCCT<br>GTGGATGACACCATTATTGGACCCCCTGGAATGCAAGTAGAAGTACTTGCTGATTCTTTACATAT<br>GCGTTTCTTAGCCCCTAAAATTGAGAATGAATACGAAACTTGGACTATGAAGAATGTGTATAACTC<br>ATGGACTTATAATGTGCAATACTGGAAAAACGGTACTGATGAAAAGTTTCAAATTACTCCCCAGTA<br>TGACTTTGAGGTCCTCAGAAACCTGGAGCCATGGACAACTTATTGTGTTCAAGTTCGAGGGTTT<br>CTTCCTGATCGGAACAAAGCTGGGGAATGGAGTGAGCCTGTCTGTGAGCAAACAACCCATGAC<br>GAAACGGTCCCCTCCTGGATGGTGGCCGTCATCCTCATGGCCTCGGTCTTCATGGTCTGCCTG<br>GCACTCCTCGGCTGCTTCGCCTTGCTGTGGTGCGTTTACAAGAAGACAAAGTACGCCTTCTCC<br>CCTAGGAATTCTCTTCCACAGCACCTGAAAGAGTTTTTGGGCCATCCTCATCATAACACACTTCT<br>GTTTTTCTCCTTTCCATTGTCGGATGAGAATGATGTTTTTGACAAGCTAAGTGTCATTGCAGAAG<br>ACTCTGAGAGCGGCAAGCAGAATCCTGGTGACAGCTGCAGCCTCGGGACCCCGCCTGGGCA<br>GGGGCCCCAAAGC
**A549 - *IFNAR2* overexpression**	**gBlocks gene fragment for *IFNAR2***	ATGCTTTTGAGCCAGAATGCCTTCATCGTCAGATCACTTAATTTGGTTCTCATGGTGTATATCAGC<br>CTCGTGTTTGGTATTTCATATGATTCGCCTGATTACACAGATGAATCTTGCACTTTCAAGATATCA<br>TTGCGAAATTTCCGGTCCATCTTATCATGGGAATTAAAAAACCACTCCATTGTACCAACTCACTAT<br>ACATTGCTGTATACAATCATGAGTAAACCAGAAGATTTGAAGGTGGTTAAGAACTGTGCAAATAC<br>CACAAGATCATTTTGTGACCTCACAGATGAGTGGAGAAGCACACACGAGGCCTATGTCACCGT<br>CCTAGAAGGATTCAGCGGGAACACAACGTTGTTCAGTTGCTCACACAATTTCTGGCTGGCCATA<br>GACATGTCTTTTGAACCACCAGAGTTTGAGATTGTTGGTTTTACCAACCACATTAATGTGATGGT<br>GAAATTTCCATCTATTGTTGAGGAAGAATTACAGTTTGATTTATCTCTCGTCATTGAAGAACAGTC<br>AGAGGGAATTGTTAAGAAGCATAAACCCGAAATAAAAGGAAACATGAGTGGAAATTTCACCTATA<br>TCATTGACAAGTTAATTCCAAACACGAACTACTGTGTATCTGTTTATTTAGAGCACAGTGATGAGC<br>AAGCAGTAATAAAGTCTCCCTTAAAATGCACCCTCCTTCCACCTGGCCAGGAATCAGAATCAGC<br>AGAATCTGCCAAAATAGGAGGAATAATTACTGTGTTTTTGATAGCATTGGTCTTGACAAGCACCA<br>TAGTGACACTGAAATGGATTGGTTATATATGCTTAAGAAATAGCCTCCCCAAAGTCTTGAGGCAA<br>GGTCTCACTAAGGGCTGGAATGCAGTGGCTATTCACAGGTGCAGTCATAATGCACTACAGTCTG<br>AAACTCCTGAGCTCAAACAGTCGTCCTGCCTAAGCTTCCCCAGTAGCTGGGATTACAAGCGTG<br>CATCCCTGTGCCCCAGTGAT

Supplementary Table 14: Nucleotide sequences.

SUPPLEMENTARY NOTES

VA Million Veteran Program COVID-19 Science Initiative: Core Acknowledgment for Publications

VA Million Veteran Program COVID-19 Science Initiative

MVP COVID-19 Science Program Steering Committee. Christopher J. O'Donnell[19], J. Michael Gaziano[19], Philip S. Tsao[24], Sumitra Muralidhar[36], Jean Beckham[37], Kyong-Mi Chang[38], Juan P. Casas[19], Kelly Cho[19,20], Saiju Pyarajan[19], Jennifer Huffman[19] & Jennifer Moser[36]

MVP COVID-19 Science Program Steering Committee Support. Lauren Thomann[19], Helene Garcon[19] & Nicole Kosik[19]

MVP COVID-19 Science Program Working Groups and Associated Chairs.

COVID-19 Related PheWAS. Katherine Liao[19] & Scott Damrauer[38]

Disease Mechanisms. Richard Hauger[39], Shiuh-Wen Luoh[28,29] & Sudha Iyengar[25-27]

Druggable Genome. Juan P. Casas[19]

Genomics for Risk Prediction, PRS, and MR. Themistocles Assimes[23,24], Panagiotis Roussos[1-7] & Robert Striker[30,31]

GWAS & Downstream Analysis. Jennifer Huffman[19] & Yan Sun[41]

Pharmacogenomics. Adriana Hung[42] & Sony Tuteja[38]

VA COVID-19 Shared Data Resource. Scott L. DuVall[21], Kristine E. Lynch[21] & Elise Gatsby[21]

MVP COVID-19 Data Core. Kelly Cho[19,20], Lauren Costa[19],Anne Yuk-Lam Ho[19] & Rebecca Song[19]

MVP COVID-19 Genomics and PRS Working Group Roster. Panagiotis Roussos[7], Tim Assimes[23,24], Rob Striker[30], Helene Garcon[19], Jenny Huffman[55], Jacob Joseph[19], Reid Thompson[29], Wen-Chih Wu[46], Austin Nguyen[17], Emily Wan[19], Renato Polimanti[43], Frank Wendt[43], Dan Levey[43], Pradeep Natarajan[47], John McGeary[46], Merry-Lynn McDonald[48], Rob Igo[25], Murray B Stein[39], Michael Lewis[49],Scott Damrauer[38], Anurag Verma[38], Kyong-Mi Chang[38], Ravi Madduri[50], Jimmy Efird[51], Darshana Jhala[38], Jeffrey Petersen[38], James Meigs[47], Benjamin Voight[52], Joel Gelernter[43], Jin Zhou[53], Michael Levin[38], Edward Siew[42], Giorgio Sirugo[54], Austin Hilliard[24], Elvis Akwo[58], Adriana Hung[42], Shiuh-Wen Luoh[28,29], Jessica Minnier[17], Rose Huang[19], Liam Gaziano[55], Sharvari Dalal[38], Cassy Robinson-Cohen[42], Ran Tao[58], Phil Tsao[24], Mehrdad Arjomandi[56], Marijana Vujkovic[38], Anoop Sendamarai[19], Uma Saxena[47] & Saiju Pyarajan[19]

MVP COVID-19 GWAS Working Group Roster. Jenny Huffman[55], Yan Sun[41], Helene Garcon[19], Jacob Joseph[19], Reid Thompson[29], Mary Wood[29], Dana Crawford[25], Emily Wan[19], Renato Polimanti[43], Frank Wendt[43], Gita Pathak[43], Pradeep Natarajan[47], John McGeary[46], Merry-Lynn McDonald[48], Rob Igo[25], Scott Damrauer[38], Anurag Verma[38], Ravi Madduri[50], Jimmy Efird[51],

Benjamin Voight[52], Joel Gelernter[43], Michael Levin[38], Richard Hauger[39], Valerio Napolioni[57], Michael Matheny[42], Alex Bick[19], Edward Siew[42], Austin Hilliard[24], Adriana Hung[42], Shiuh-Wen Luoh[28,29,40], Rose Huang[19], Matt Freiberg[58], Quinn S. Wells[58], Cassy Robinson-Cohen[42], Alex Pereira[40], Kyong-Mi Chang[38], Saiju Pyarajan[19], Anoop Sendamarai[19], Uma Saxena[47], Gina Peloso[55] & Catherine Tcheandjieu[24]

MVP Executive Committee. J. Michael Gaziano[19] , Sumitra Muralidhar[36], Rachel Ramoni[36] , Jean Beckham[37], Kyong-Mi Chang[38], Christopher J. O'Donnell[19], Philip S. Tsao[24], James Breeling[36], Grant Huang[36] & Juan P. Casas[19]

MVP Program Office. Sumitra Muralidhar[36] & Jennifer Moser[36]

MVP Recruitment/Enrollment.

Director/Deputy Director, Boston. Stacey B. Whitbourne[19] & Jessica V. Brewer[19]

Clinical Epidemiology Research Center (CERC), West Haven. Mihaela Aslan[43]

Cooperative Studies Program Clinical Research Pharmacy Coordinating Center, Albuquerque. Todd Connor[44] & Dean P. Argyres[44]

Genomics Coordinating Center, Palo Alto. Philip S. Tsao[24]

MVP Boston Coordinating Center, Boston. J. Michael Gaziano[19]

MVP Information Center, Canandaigua. Brady Stephens[45]

VA Central Biorepository, Boston. Mary T. Brophy[19], Donald E. Humphries[19] & Luis E. Selva[19]

MVP Informatics, Boston. Nhan Do[19] & Shahpoor (Alex) Shayan[19]

MVP Data Operations/Analytics, Boston. Kelly Cho[19,20]

Director of Regulatory Affairs. Lori Churby[24]

MVP Science.

Science Operations. Christopher J. O'Donnell[19]

Genomics Core. Christopher J. O'Donnell[19], Saiju Pyarajan[19] & Philip S. Tsao[24]

Data Core. Kelly Cho[19,20]

VA Informatics and Computing Infrastructure (VINCI). Scott L. DuVall[21]

Data and Computational Sciences. Saiju Pyarajan[19]

Statistical Genetics. Elizabeth Hauser[37] , Yan Sun[41] & Hongyu Zhao[43]

MVP Local Site Investigators. Peter Wilson[41], Rachel McArdle[59], Louis Dellitalia[60], Kristin Mattocks[61], John Harley[62], Jeffrey Whittle[63], Frank Jacono[27], Jean Beckham[37], John Wells[64], Salvador Gutierrez[65], Gretchen Gibson[66], Kimberly Hammer[67], Laurence Kaminsky[68], Gerardo Villareal[44], Scott Kinlay[19], Junzhe Xu[69], Mark Hamner[70], Roy Mathew[71], Sujata Bhushan[72], Pran Iruvanti[73], Michael Godschalk[74], Zuhair Ballas[75], Douglas Ivins[76], Stephen Mastorides[77], Jonathan Moorman[78], Saib Gappy[79], Jon Klein[80], Nora Ratcliffe[81], Hermes Florez[82], Olaoluwa Okusaga[83], Maureen Murdoch[84], Peruvemba Sriram[85], Shing Shing Yeh[86], Neeraj Tandon[87], Darshana Jhala[38], Samuel Aguayo[88], David Cohen[29], Satish Sharma[46], Suthat Liangpunsakul[89], Kris Ann Oursler[90], Mary Whooley[91], Sunil Ahuja[92], Joseph Constans[93], Paul Meyer[94], Jennifer Greco[95], Michael Rauchman[96], Richard Servatius[97], Melinda Gaddy[98], Agnes Wallbom[49], Timothy

Morgan[99], Todd Stapley[100], Scott Sherman[101], George Ross[102], Philip Tsao[24], Patrick Strollo[103], Edward Boyko[104], Laurence Meyer[105], Samir Gupta[39], Mostaqul Huq[106], Joseph Fayad[107], Adriana Hung[42], Jack Lichy[108], Robin Hurley[109], Brooks Robey[110] & Robert Striker[30,31]

[1]Department of Psychiatry, Icahn School of Medicine at Mount Sinai, New York, NY, USA. [2]Center for Disease Neurogenomics, Icahn School of Medicine at Mount Sinai, New York, NY, USA. [3]Pamela Sklar Division of Psychiatric Genomics, Icahn School of Medicine at Mount Sinai, New York, NY, USA. [4]Friedman Brain Institute, Icahn School of Medicine at Mount Sinai, New York, NY, USA. [5]Department of Genetics and Genomic Science, Icahn School of Medicine at Mount Sinai, New York, NY, USA. [6]Icahn Institute for Data Science and Genomic Technology, Icahn School of Medicine at Mount Sinai, New York, NY, USA. [7]Mental Illness Research, Education, and Clinical Center (VISN 2 South), James J. Peters VA Medical Center, Bronx, NY, USA. [8]Nash Family Department of Neuroscience, Icahn School of Medicine at Mount Sinai, New York, NY, USA. [9]Department of Psychiatry, Yale University, New Haven, CT, USA. [10]Mount Sinai Clinical Intelligence Center, Icahn School of Medicine at Mount Sinai, New York, NY, USA. [11]Department of Microbiology, Grossman School of Medicine, New York University, New York, NY, USA. [12]Department of Medicine, Grossman School of Medicine, New York University, New York, NY, USA. [13]Vilcek Institute of Graduate Biomedical Sciences, New York University, New York, NY, USA. [14]Department of Microbiology, Icahn School of Medicine at Mount Sinai, New York, NY, USA. [15]Virus Engineering Center for Therapeutics and Research, Icahn School of Medicine at Mount Sinai, New York, NY, USA. [16]Global Health and Emerging Pathogens Institute, Icahn School of Medicine at Mount Sinai, New York, NY, USA. [17]Biostatistics Shared Resources, Knight Cancer Institute, Oregon Health & Science University, Portland, OR, USA. [18]New York Genome Center, New York, NY, USA. [19]VA Boston Healthcare System, Boston, MA, USA. [20]Division of Aging, Brigham and Women's Hospital, Harvard Medical School, Boston, MA, USA. [21]VA Informatics and Computing Infrastructure, VA Salt Lake City Health Care System, Salt Lake City, UT, USA. [22]Division of Epidemiology, University of Utah, Salt Lake City, UT, USA. [23]Department of Medicine, Stanford University School of Medicine, Stanford, CA, USA. [24]VA Palo Alto Health Care System, Palo Alto, CA, USA. [25]Department of Population and Quantitative Health Sciences, School of Medicine, Case Western Reserve University, Cleveland, OH, USA. [26]Department of Genetics and Genome Sciences, School of Medicine, Case Western Reserve University, Cleveland, OH, USA. [27]VA Northeast Ohio Healthcare System, Cleveland VA Medical Center, Cleveland, OH, USA. [28]Department of Medicine, Knight Cancer Institute, Oregon Health & Science University, Portland, OR, USA. [29]VA Portland Health Care System, Portland, OR, USA. [30]Division of Infectious Diseases, Department of Medicine, University of Wisconsin, Madison, WI, USA. [31]William S. Middleton Memorial Veterans Hospital, Madison, WI, USA. [32]Sema4, Stamford, CT, USA. [33]Department of Oncological Sciences, Icahn School of Medicine at Mount Sinai, New York, NY, USA. [34]Precision Immunology Institute, Icahn School of Medicine at Mount Sinai, New York, NY, USA. [35]Tisch Cancer Institute, Icahn School of Medicine at Mount Sinai, New York, NY, USA. [36]US Department of Veterans Affairs, Washington, DC, USA. [37]Durham VA Medical Center, Durham, NC, USA. [38]Corporal Michael J Crescenz VA Medical Center, Philadelphia, PA, USA. [39]VA San Diego Healthcare System, San Diego, CA, USA. [40]Department of Genetics, Harvard Medical School, Harvard University, Boston, MA, USA. [41]Atlanta VA Medical Center, Decatur, GA, USA. [42]VA Tennessee Valley Healthcare System,

South Nashville, TN, USA. [43]VA Connecticut Healthcare System, West Haven, CT, USA. [44]New Mexico VA Health Care System, Albuquerque, NM, USA. [45]Canandaigua VA Medical Center, Canandaigua, NY, USA. [46]Providence VA Medical Center, Providence, RI, USA. [47]Massachusetts General Hospital, Boston, MA, USA. [48] Division of Pulmonary, Allergy and Critical Care Medicine, University of Alabama at Birmingham, Birmingham, AL, USA . [49]VA Greater Los Angeles Healthcare System, Los Angeles, CA, USA. [50]Argonne National Laboratory, Chicago, IL, USA . [51]VA Cooperative Studies Program Coordinating Center, Boston, MA, USA. [52]Institute of Diabetes Obesity and Metabolism, University of Pennsylvania, Philadelphia, PA, USA. [53]UCLA Medical School, Los Angeles, CA, USA. [54]Division of Translational Medicine and Human Genetics, Department of Medicine, University of Pennsylvania School of Medicine, Philadelphia, PA, USA. [55]Massachusetts Veterans Epidemiology Research and Information Center (MAVERIC), Cardiology Section, VA Boston Healthcare System, Boston, MA, USA. [56]UCSF Medical Center, Medicine, San Francisco, CA, USA. [57]Department of Neurology and Neurological Sciences, Stanford University School of Medicine, Stanford, CA, USA. [58]Vanderbilt University Medical Center, Nashville, TN, USA. [59]Bay Pines VA Healthcare System, Bay Pines, FL, USA. [60]Birmingham VA Medical Center, Birmingham AL, USA. [61]Central Western Massachusetts Healthcare System, Leeds, MA, USA. [62]Cincinnati VA Medical Center, Cincinnati, OH, USA. [63]Clement J. Zablocki VA Medical Center, Milwaukee, WI, USA. [64]Edith Nourse Rogers Memorial Veterans Hospital, Bedford, MA, USA
[65]Edward Hines, Jr. VA Medical Center, Hines, IL, USA. [66]Veterans Health Care System of the Ozarks, Fayetteville, AR, USA. [67]Fargo VA Health Care System, Fargo, ND, USA. [68]VA Health Care Upstate New York, Albany, NY, USA. [69]VA Western New York Healthcare System, Buffalo, NY, USA. [70]Ralph H. Johnson VA Medical Center, Mental Health Research, Charleston, SC, USA. [71]Columbia VA Health Care System, Columbia, SC, USA. [72]VA North Texas Health Care System, Dallas, TX, USA. [73]Hampton VA Medical Center, Hampton, VA, USA. [74]Richmond VA Medical Center, Richmond, VA, USA. [75]Iowa City VA Health Care System, Iowa City, IA, USA. [76]Eastern Oklahoma VA Health Care System, Muskogee, OK, USA. [77]James A. Haley Veterans' Hospital, Tampa, FL, USA. [78]James H. Quillen VA Medical Center, Mountain Home, TN, USA. [79]John D. Dingell VA Medical Center, Detroit, MI, USA. [80]Louisville VA Medical Center, Louisville, KY, USA. [81]Manchester VA Medical Center, Manchester, NH, USA. [82]Miami VA Health Care System, Miami FL, USA. [83]Michael E. DeBakey VA Medical Center, Houston, TX, USA. [84]Minneapolis VA Health Care System, Minneapolis, MN, USA. [85]N. FL/S. GA Veterans Health System, Gainesville, FL, USA. [86]Northport VA Medical Center, Northport, NY, USA. [87]Overton Brooks VA Medical Center, Shreveport, LA, USA. [88]Phoenix VA Health Care System, Phoenix, AZ, USA. [89]Richard Roudebush VA Medical Center, Indianapolis, IN, USA. [90]Salem VA Medical Center, Salem, VA, USA. [91]San Francisco VA Health Care System, San Francisco, CA, USA. [92]South Texas Veterans Health Care System, San Antonio, TX, USA. [93]Southeast Louisiana Veterans Health Care System, New Orleans, LA, USA. [94]Southern Arizona VA Health Care System, Tucson, AZ, USA. [95]Sioux Falls VA Health Care System, Sioux Falls, SD, USA. [96]St. Louis VA Health Care System, St. Louis, MO, USA. [97]Syracuse VA Medical Center, Syracuse, NY, USA. [98]VA Eastern Kansas Health Care System, Leavenworth, KS, USA. [99]VA Long Beach Healthcare System, Long Beach, CA, USA. [100]VA Maine Healthcare System, Augusta, ME, USA. [101]VA New York Harbor Healthcare System, New York, NY, USA. [102]VA Pacific Islands Health Care System, Honolulu, HI, USA. [103]VA Pittsburgh Health Care System, Pittsburgh, PA,

USA. [104]VA Puget Sound Health Care System, Seattle, WA, USA. [105]VA Salt Lake City Health Care System, Salt Lake City, UT, USA. [106]VA Sierra Nevada Health Care System, Reno, NV, USA. [107]VA Southern Nevada Healthcare System, North Las Vegas, NV, USA. [108]Washington DC VA Medical Center, Washington, D. C., USA. [109]W.G. (Bill) Hefner VA Medical Center, Salisbury, NC, USA & [110]White River Junction VA Medical Center, White River Junction, VT, USA

Mount Sinai COVID-19 Biobank: Core Acknowledgment for Publications

Charuta Agashe[111], Priyal Agrawal[111], Alara Akyatan[111], Kasey Alesso-Carra[111], Eziwoma Alibo[111], Kelvin Alvarez[111], Angelo Amabile[111], Carmen Argmann[111], Kimberly Argueta[111], Steven Ascolillo[111], Rasheed Bailey[111], Craig Batchelor[111], Noam D. Beckmann[5,6,10,111], Aviva G. Beckmann[111], Priya Begani[111], Jessica Le Berichel[111], Dusan Bogunovic[111], Swaroop Bose[111], Cansu Cimen Bozkus[111], Paloma Bravo[111], Mark Buckup[111], Larissa Burka[111], Sharlene Calorossi[111], Lena Cambron[111], Guillermo Carbonell[111], Gina Carrara[111], Mario A. Cedillo[111], Christie Chang[111], Serena Chang[111], Alexander W. Charney[1,3,5,6,8,10,111], Steven T. Chen[111], Esther Cheng[111], Jonathan Chien[111], Mashkura Chowdhury[111], Jonathan Chung[111], Phillip H. Comella[111], Dana Cosgrove[111], Francesca Cossarini[111], Liam Cotter[111], Arpit Dave[111], Travis Dawson[111], Bheesham Dayal[111], Diane Marie Del Valle[111], Maxime Dhainaut[111], Rebecca Dornfeld[111], Katie Dul[111], Melody Eaton[111], Nissan Eber[111], Cordelia Elaiho[111], Ethan Ellis[111], Frank Fabris[111], Jeremiah Faith[111], Dominique Falci[111], Susie Feng[111], Brian Fennessy[111], Marie Fernandes[111], Nataly Fishman[111], Nancy J. Francoeur[111], Sandeep Gangadharan[111], Daniel Geanon[111], Bruce D. Gelb[111], Benjamin S. Glicksberg[111], Sacha Gnjatic[111], Joanna Grabowska[111], Gavin Gyimesi[111], Maha Hamdani[111], Diana Handler[111], Jocelyn Harris[111], Matthew Hartnett[111], Sandra Hatem[111], Manon Herbinet[111], Elva Herrera[111], Arielle Hochman[111], Gabriel E. Hoffman[1-6,111], Jaime Hook[111], Laila Horta[111], Etienne Humblin[111], Suraj Jaladanki[111], Hajra Jamal[111], Jessica S. Johnson[111], Gurpawan Kang[111], Neha Karekar[111], Subha Karim[111], Geoffrey Kelly[111], Jong Kim[111], Seunghee Kim-Schulze[111], Arvind Kumar[111], Jose Lacunza[111], Alona Lansky[111], Dannielle Lebovitch[111], Brian Lee[111], Grace Lee[111], Gyu Ho Lee[111], Jacky Lee[111], John Leech[111], Lauren Lepow[111], Michael B. Leventhal[111], Lora E. Liharska[111], Katherine Lindblad[111], Alexandra Livanos[111], Bojan Losic[111], Rosalie Machado[111], Kent Madrid[111], Zafar Mahmood[111], Kelcey Mar[111], Thomas U. Marron[111], Glenn Martin[111], Robert Marvin[111], Shrisha Maskey[111], Paul Matthews[111], Katherine Meckel[111], Saurabh Mehandru[111], Miriam Merad[33-35,111], Cynthia Mercedes[111], Elyze Merzier[111], Dara Meyer[111], Gurkan Mollaoglu[111], Sarah Morris[111], Konstantinos Mouskas[111], Emily Moya[111], Naa-akomaah Yeboah[111], Girish Nadkarni[111], Kai Nie, Marjorie Nisenholtz[111], George Ofori-Amanfo[111], Kenan Onel[111], Merouane Ounadjela[111], Manishkumar Patel[111], Vishwendra Patel[111], Cassandra Pruitt[111], Adeeb Rahman[111], Shivani Rathi[111], Jamie Redes[111], Ivan Reyes-Torres[111], Alcina Rodrigues[111], Alfonso Rodriguez[111], Vladimir Roudko[111], Panos Roussos[1-7,111], Evelyn Ruiz[111], Pearl Scalzo, Eric E. Schadt[5,6,32,111], Ieisha Scott[111], Robert Sebra[111], Hardik Shah[111], Pedro Silva[111], Nicole W. Simons[111], Melissa Smith[111], Alessandra Soares Schanoski[111], Juan Soto[111], Shwetha Hara Sridhar[111], Stacey-Ann Brown[111], Hiyab Stefanos[111], Meghan Straw[111], Robert Sweeney[111], Alexandra Tabachnikova[111], Collin Teague[111], Ryan Thompson[111], Manying Tin[111], Kevin Tuballes[111], Scott R. Tyler[111], Bhaskar Upadhyaya[111], Akhil Vaid[111], Verena Van Der Heide[111], Natalie Vaninov[111], Konstantinos Vlachos[111], Daniel

Wacker[111], Laura Walker[111], Hadley Walsh[111], Bo Wang[111], Wenhui Wang[111], C. Matthias Wilk[111], Lillian Wilkins[111], Karen M. Wilson[111], Jessica Wilson[111], Hui Xie[111], Li Xue[111], Nancy Yi[111], Ying-chih Wang[111], Mahlet Yishak[111], Sabina Young[111], Alex Yu[111], Nina Zaks[111] & Renyuan Zha[111]

[1]Department of Psychiatry, Icahn School of Medicine at Mount Sinai, New York, NY, USA. [2]Center for Disease Neurogenomics, Icahn School of Medicine at Mount Sinai, New York, NY, USA. [3]Pamela Sklar Division of Psychiatric Genomics, Icahn School of Medicine at Mount Sinai, New York, NY, USA. [4]Friedman Brain Institute, Icahn School of Medicine at Mount Sinai, New York, NY, USA. [5]Department of Genetics and Genomic Science, Icahn School of Medicine at Mount Sinai, New York, NY, USA. [6]Icahn Institute for Data Science and Genomic Technology, Icahn School of Medicine at Mount Sinai, New York, NY, USA. [7]Mental Illness Research, Education, and Clinical Center (VISN 2 South), James J. Peters VA Medical Center, Bronx, NY, USA. [8]Nash Family Department of Neuroscience, Icahn School of Medicine at Mount Sinai, New York, NY, USA. [10]Mount Sinai Clinical Intelligence Center, Icahn School of Medicine at Mount Sinai, New York, NY, USA. [32]Sema4, Stamford, CT, USA. [33]Department of Oncological Sciences, Icahn School of Medicine at Mount Sinai, New York, NY, USA. [34]Precision Immunology Institute, Icahn School of Medicine at Mount Sinai, New York, NY, USA. [35]Tisch Cancer Institute, Icahn School of Medicine at Mount Sinai, New York, NY, USA. [111]Mount Sinai COVID-19 Biobank, Icahn School of Medicine at Mount Sinai, New York, NY, USA.

Other Acknowledgements

Figure 4b: Created with BioRender.com.

DESCRIPTION OF THE SUPPLEMENTARY DATA FILES

SUPPLEMENTARY DATA 1 TO 7

Brief description: TWAS results for COVID-19 GWASs:

Name	Phenotype	TWAS results in
A1	Very severe respiratory confirmed covid vs. not hospitalized covid	Supplementary Data 1
A2	Very severe respiratory confirmed COVID vs. population	Supplementary Data 2
B1	Hospitalized COVID vs. not hospitalized COVID	Supplementary Data 3
B2	Hospitalized COVID vs. population	Supplementary Data 4
C1	COVID vs. lab/self-reported negative	Supplementary Data 5
C2	COVID vs. population	Supplementary Data 6
D1	predicted COVID from self-reported symptoms vs. predicted or self-reported non-COVID	Supplementary Data 7

Each sheet within an excel file represents a different tissue. Column descriptions:

Column	Description
gene	a gene's id: as listed in the Tissue Transcriptome model. Ensemble Id for most gene model releases (e.g. in this study). Can also be an intron's id for splicing model releases
gene_name	gene name as listed by the Transcriptome Model, typically HUGO for a gene (e.g. in this study). It can also be an intron's id.
zscore	S-PrediXcan or S-EpiXcan's association result for the gene
effect_size	S-PrediXcan or S-EpiXcan's association effect size for the gene. Can only be computed when beta from the GWAS is used.
pvalue	p-value of the aforementioned statistic
var_g	variance of the gene expression, calculated as W' * G * W (where W is the vector of SNP weights in a gene's model, W' is its transpose, and G is the covariance matrix)
pred_perf_r2	R^2_{CV} (cross-validated) of tissue model's correlation to gene's measured transcriptome (prediction performance). Recommended filtering is > 0.01
pred_perf_pval	p-value of tissue model's correlation to gene's measured transcriptome (prediction performance).
pred_perf_qval	q-value of tissue model's correlation to gene's measured transcriptome (prediction performance).
n_snps_used	number of SNPs from GWAS that were used in the S-PrediXcan or S-EpiXcan analysis
n_snps_in_cov	number of SNPs in the covariance matrix
n_snps_in_model	number of SNPs in the imputation model
gwas	GWAS name (phenotype) from the COVID-19 hg
tissue	Imputation model used
method	Imputation method used for imputation model construction (in this study PrediXcan or EpiXcan)
gwas_fdr	FDR-adjusted p value[2] for association when considering all gene-trait associations across all tissue models within this specific GWAS (e.g. COVID-19 B2 phenotype)

Tissue.keyword	Pattern matching string for internal pipeline
fdr_all	FDR-adjusted p value[2] for association when considering all gene-trait associations across all tissue models and all GWASs (all COVID-19 phenotypes)
var_g	variance of the gene expression, calculated as W' * G * W (where W is the vector of SNP weights in a gene's model, W' is its transpose, and G is the covariance matrix)

SUPPLEMENTARY DATA 8

Brief description: Results from JEPEGMIX2-P pathway analysis.

Column descriptions (Sheet: Summary_of_significant):

Column	Description
Name	Gene set name
Count.Bon.Sig	Number of tissues were this gene set is Bonferroni significant
Min.Pval	Min JEPEGMIX2-P p-value from all tissues
Min.Bonferroni	Min Bonferroni-adjusted JEPEGMIX2-P p-value from all tissues

Column descriptions (Sheet: All_results):

Column	Description
Type	Type name
Tissue	Tissue name
Name	Gene set name
df	Degrees of freedom
Chisq	chi-square
Pval	JEPEGMIX2-P p-value
Qval_holm	JEPEGMIX2-P q-value with Holm method
Qval_fdr	JEPEGMIX2-P q-value with FDR method
Dom_sing	JEPEGMIX2-P dominate signal
Sign_genes	JEPEGMIX2-P significant genes

SUPPLEMENTARY DATA 9

Brief description: Results of *IL10RB* and *IFNAR2* GReX PheWAS

Column descriptions:

Column	Description
ID	Concatenation of gene and phenotype analyzed
phecode	Phecode identifier in string format
beta	Association of gene expression and phenotype for gene and phenotype described in ID column
p	P value for association of gene expression and phenotype for gene and phenotype described in ID column
neg_log10p	$-\log_{10}$ transformation of p column
beta_dir	Binary classifier for direction of beta column. TRUE if positive. FALSE if negative.
beta_mag	Absolute value of beta column
phecode_num	Numerical phecode
Phenotype	Phenotype associated with each phecode
exclude_name	Category for each phecode. Phecodes were grouped into categories using Phecode Map v1.2 with manual curation for some uncategorized phecodes. Refer to Supplementary Data 10 for mappings.
HasCounts	Number of individuals in cohort with >0 counts for this phecode
NoCounts	Number of individuals in cohort with 0 counts for this phecode
gene	Gene whose expression was used in regression model for the specified phecode
adjusted.p	Adjusted p value using method specified in MC.method column
neg_log10adjusted.p	$-\log_{10}$ transformation of adjusted.p column
Rank	Rank of association significance from most significant to least
MC.method	Method for generating adjusted.p column from p column

SUPPLEMENTARY DATA 10

Brief description: Table translating Phecodes to Phenotypes and the respective phenotype categories they belong to.

Column descriptions:

Column	Description
Phecode	Numerical phecode
Phenotype	Phenotype associated with phecode
exclude_name	Category for each phecode. Phecodes were grouped into categories using Phecode Map v1.2 with manual curation for some uncategorized phecodes

SUPPLEMENTARY RESULTS

COVID-19 phenotypes genetically regulated gene expression (GReX) comparison.

As shown by correlation, hierarchical and principal component analysis (Supplementary Fig. 2) of the COVID-19 phenotypes GReX, the phenotypes mainly cluster in 4 groups: *(1)* The severe vs. not severe COVID group (A1 and B1), *(2)* The severe COVID vs. population group (A2 and B2), *(3)* the any COVID vs. population or lab/self-reported negative group (C1 and C2) and finally *(4)* a group comprising the predicted COVID phenotype (D1). It is worth noting that this GReX-based phenotypic clustering persists, despite differences in the different ancestries included in the genetic analysis (e.g. A1&B1, A2&B2, C1&C2) (Supplementary Table 2).

SUPPLEMENTARY REFERENCES

1. Del Valle, D. M. *et al.* An inflammatory cytokine signature predicts COVID-19 severity and survival. *Nat. Med.* **26**, 1636–1643 (2020).
2. Hochberg, Y. & Benjamini, Y. More powerful procedures for multiple significance testing. *Stat. Med.* **9**, 811–818 (1990).
